# The role of interdisciplinary research team in the impact of health apps in health and computer science publications: a systematic review

**DOI:** 10.1186/s12938-016-0185-y

**Published:** 2016-07-15

**Authors:** Guillermo Molina Recio, Laura García-Hernández, Rafael Molina Luque, Lorenzo Salas-Morera

**Affiliations:** 1 Nursing Department, University of Córdoba, Córdoba, Spain; 2 Area of Project Engineering, University of Córdoba, Córdoba, Spain

## Abstract

**Background:**

Several studies have estimated the potential economic and social impact of the mHealth development. Considering the latest study by Institute for Healthcare Informatics, more than 165.000 apps of health and medicine are offered including all the stores from different platforms. Thus, the global mHealth market was an estimated $10.5 billion in 2014 and is expected to grow 33.5 percent annually between 2015 and 2020s. In fact, apps of Health have become the third-fastest growing category, only after games and utilities.

**Methods:**

This study aims to identify, study and evaluate the role of interdisciplinary research teams in the development of articles and applications in the field of mHealth. It also aims to evaluate the impact that the development of mHealth has had on the health and computer science field, through the study of publications in specific databases for each area which have been published until nowadays.

**Results:**

Interdisciplinary nature is strongly connected to the scientific quality of the journal in which the work is published. This way, there are significant differences in those works that are made up by an interdisciplinary research team because of they achieve to publish in journals with higher quartiles. There are already studies that warn of methodological deficits in some studies in mHealth, low accuracy and no reproducibility. Studies of low precision and poor reproducibility, coupled with the low evidence, provide low degrees of recommendation of the interventions targeted and therefore low applicability.

**Conclusions:**

From the evidence of this study, working in interdisciplinary groups from different areas greatly enhances the quality of research work as well as the quality of the publications derived from its results.

## Background

Several studies have estimated the potential economic and social impact of the mHealth development. mHealth is an abbreviation for mobile health, a term used for the practice of medicine and public health supported by mobile devices. According to WHO [1], nearly 90 % of the world population could benefit from the opportunities offered by mobile technologies and with a relatively low cost. Considering the latest study by Institute for Healthcare Informatics (IMS) [2], more than 165.000 apps of health and medicine are offered including all the stores from different platforms. Thus, the global mHealth market was an estimated $10.5 billion in 2014 and is expected to grow 33.5 percent annually between 2015 and 2020s [3].

Also, the IMS Institute indicates that 70 % of health apps is focused on general population, offering tools to reach and maintain wellness and to improve physical activity. The remaining 30 %, were designed to more concrete areas such as professionals or people affected by specific diseases.

Despite this situation, it is important to note that more than 50 % of the available apps received less than 500 downloads and only five of them comprise 15 % of all those in the health category. The IMS attributed this situation to different causes, which include: poor quality in many of them, the lack of guidance on the usefulness of the app and a low level of support from health professionals.

However, it is well-known that health apps, solving the problems detailed above, could represent a very useful tool for monitoring chronic diseases will account for 65 % of the global market for mHealth in 2017 [3].

This fact will represent revenue of 15.000 million dollars. The pathologies with a higher potential to increase business are in order: diabetes and cardiovascular disease. They will also play an important role related to diagnostic services (they will reach 15 % and will generate 3.400 million of dollars) and medical treatments (10 % of the market and revenues of 2.300 million). By the other hand, it is estimated that business will increase from 4.500 million in 2013, to 23.000 million in 2017. Continents with largest market share are, in descending order, Europe and Asia (30 %), United States of America and Canada (28 %) [3].

However, we do not know if the apps available to the population are based on scientific knowledge and therefore, it is difficult to assess the real impact of this spectacular development on the health of populations. On the other hand, we do not know how the great spread of the phenomenon of Health 2.0 (that is a *term presented in the mid*-*2000s, as the subset of health technologies mirroring the wider Web 2.0 movement, offering possibilities for changing health care which started with the introduction of eHealth following the emergence of the World Wide Web* [4, 5] ) that is reaching the scientific field (medical or computer), which should occur in parallel in order to offer products that positively affect the health of citizens.

Therefore, this study aims to identify, study and evaluate the role of interdisciplinary research teams in the development of articles and applications in the field of mHealth and the impact that the development of mHealth has had on the health and computer science field, through the study of publications and the composition of the research teams in specific databases for each area, which have been published until nowadays. According to Yadros et al. [6] interdisciplinary research seems to be a supplier of creative and innovative approaches. It is able to produce new lines of research and renew scientific field. In this sense, the justification of using interdisciplinary research is thus particularly strong and crucial in scientific programmes addressing grand societal issues or challenges that require an holistic approach including biological, physical and social factors.

## Methods

**Fig. 1:**
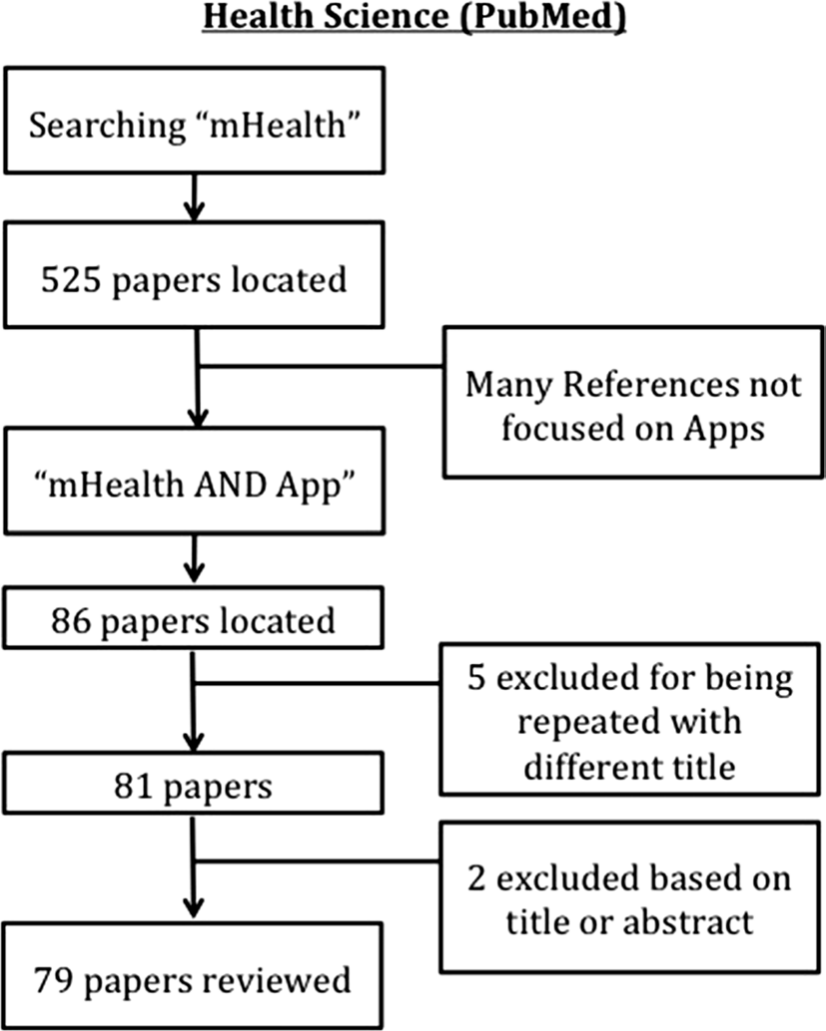
Review and selection criteria of papers (health science)

**Fig. 2:**
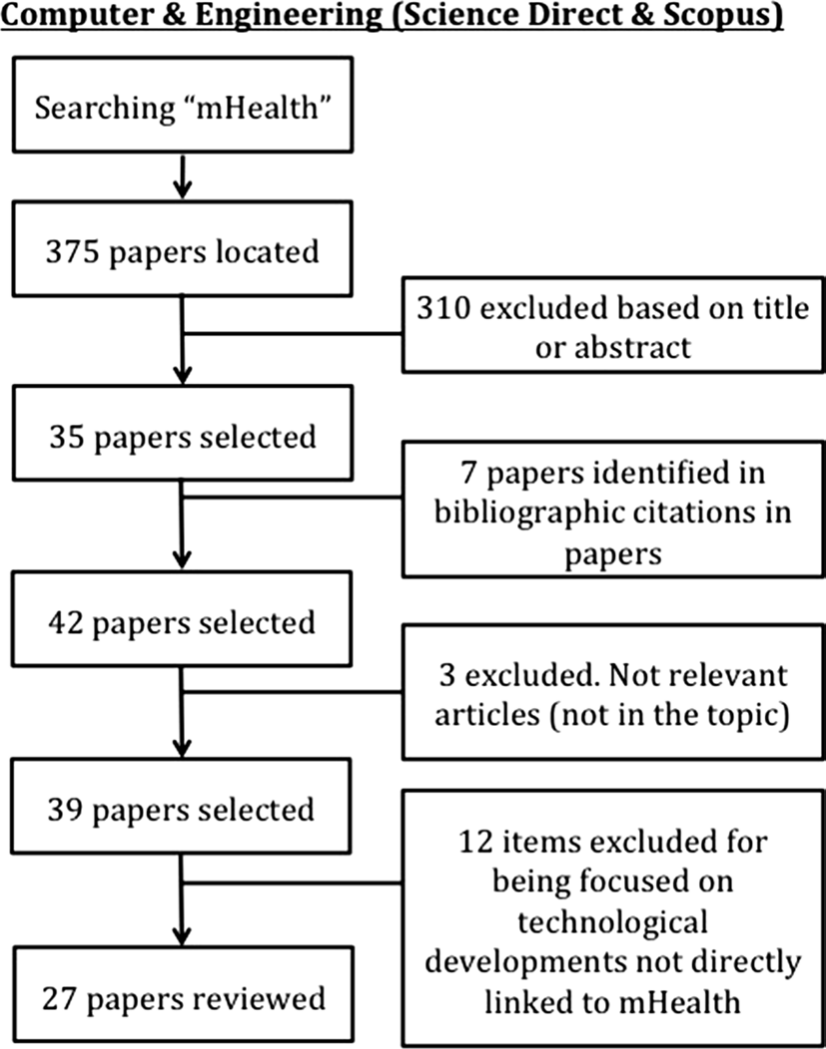
Review and selection criteria of papers (computer science)

This work is extended and based on the previous work [7]. A systematic review was conducted in two stages during November 2014. The first one was focused on locating papers available in databases from Health Sciences. After this step, we repeat the search but in the Computer Science field because we wanted to find the different penetration in each area. As recommended in the PRISMA Statement [8] for systematic reviews. The PRISMA Statement consists of a 27-item checklist and a four-phase flow diagram. The aim of the PRISMA Statement is to help authors improve the reporting of systematic reviews and meta-analyses. We describe the search strategy and the number of papers located, discarded and finally selected for review using a for-phase flow diagram. In the first stage, we have consulted the PubMed database. We used “mHealth” and “mHealth AND app” terms as search strategies. Finally 79 items were selected for reviewing (see Fig. 1). We also consulted “Science Direct” and “Scopus” using “mHealth” but reducing the search to “Computer Science” area. Having initially located 375 publications, only 27 were chosen for our study (see Fig. 2). Thus, a total of 106 items were reviewed. The impact factor of the journals that published the papers selected was consulted using the journal citation report from web of science (WoS). Since this impact factor usually varies for each year, we took the corresponding to the year when the article was published.

As noted previously, the execution of this work has been carried out by means of the recommendations given by the PRISMA [8] statement. So, this work includes a study through the following information:

- The summaries and results of all reviewed papers after performing his complete reading.
- The departments that participate in the development of the works and their categorization for subsequent statistical analysis.

This last categorization has been performed including the departments in ten large groups, which are: ‘Research Center’, ‘Nursing and other health professionals’, ‘Engineering and Technology’, ‘Finance and Statistics’, ‘General Medicine and Specialties’, ‘Agencies and Institutions’, ‘Health’, ‘Health Care and Community’, ‘Physiotherapy Associates’, ‘Pharmacy and Associates’.

In order to analyze and evaluate the impact of health apps in health and computer science publications in a precise way, additional features which are not used to be considered into systematic reviews, have been included in this study. All the information taken into account has been clustered into two main categories:

1. Publication characteristics:
  Journal name.
  Type of journal (According to 22 categories taken from ISI Web of Knowledge).
  Journal ranking (quartile).
  Journal impact factor.
  Article publication date.
  Type of study.
  Number of received citations.
2. Interdisciplinary nature:
  Departments working on the contribution.

The evaluation of the last item (interdisciplinary nature) has been performed using the Rao–Stirling index as explained by Rafolds and Meyer [9], among others. Interdisciplinary research has been defined as a mode of research that integrates techniques, tools, and/or theories from two or more disciplines to advance fundamental understanding or to solve problems whose solutions are beyond the scope of a single discipline or area of research practice [10]. The advantage of the Rao–Stirling measure is that it takes into account the distribution of references across disciplinary categories on journals in the WoS for 220 WoS categories or subject categories (SC) and also considers how cognitively distant these categories are.

For statistical analysis of the data we have used own descriptive statistics (frequency tables, measures of central tendency and dispersion, Pearson correlation coefficient as well as graphic representation) and analytical techniques, using as evidence contrast hypothesis Chi square, t Student, ANOVA and Kruskal–Wallis as non-parametric version. Processing and analysis of data was performed using the SPSS version 22.0.0 [11].

## Results and discussion

A brief summary of the main features, topics and contents in the reviewed papers is shown in Tables 1 and 2. We found papers focused on mHealth in 51 different journals, being most of them (68.6 %) indexed in the ISI web of Knowledge. The higher proportion of papers published in journal indexed in JCR, is located in four WoS categories: medical informatics (17.9 %), Healthcare Sciences & Services (12.8 %), computer science interdisciplinary applications (11.1 %) and mathematical and computational biology (8.5 %), showing a great concern about developing mHealth research in two fields of ISI that we think that must be strongly linked to this topic: clinical medicine and computer science. Taking into account not indexed journals, we found same development patterns, because most of the researches belong to different departments and institutions, including professionals from health and computer science. This fact could be explained by the concentration of articles in just three journals, journal of medical internet research mHealth and uHealth, (JMU) containing 21.7 % of the published papers, journal of medical internet research (JMIR), covering 7.5 % and international journal of medical informatics, reaching 4.7 % papers. Thus, for example, journal of medical internet research is classified by journal citation report (JCR) into several categories (“Health Care Sciences & Services” and “Medical Informatics”) and the international journal of medical informatics is listed by JCR into three categories, the two previously mentioned, as well as in “Computer Science, Information Systems” that clearly belongs to a non-health area. Another important result is the great impact factor of these publications. This way, in any category where they could be classified of these two journals belong to the first quartile, excepting JMU which is not indexed because it was created in 2013 like an spin-off from JMIR. This may also explain that the average impact factor of the found publications was 1.54 (±1898) including all the papers, and 3.0248 (±1.6014) if we select just the papers published in indexed journals. Moreover, 27.4 % of the articles are located in first quartile journals, 13.2 % in the second, 7.5 % in the third and 2.8 % in the fourth quartile. The 49.1 % of the papers appear in not indexed journals, specially in JMU. From our point of view, this fact highlights the high impact and newness that scientific work based on the use of mHealth technology represents for editors, allowing researchers to access to high impact publications and how new categories of are opening progressively.

**Table 1:** Main features of reviewed papers

Authors/year	Type of study	Journal	Type of journal	Quartile	Impact factor	Number citations	Departments involved
Brown et al. [42]	Qualitative research	Journal of biomedical informatics	Computer science	Q1	2.482	7	1. HIV Center for Clinical and Behavioral Studies 2. Department of Biomedical Informatics 3. Department of Biomedical Informatics 4. School of Nursing
Balsam et al. [43]	Technological improvement	Sensors and actuators B: chemical	Chemistry	Q1	3.840	2	1. Division of Biology, Office of Science and Engineering 2. University of Maryland 3. Division of Cancer Biology, National Cancer Institute
Akter et al. [44]	Qualitative research	Information and management	Social science, general	Q1	1.788	8	1. School of Management and Marketing 2. Australian School of Business
Van der Heijden et al. [31]	Technological development	Journal of biomedical informatics	Computer science	Q1	2.482	11	1. Institute for Computing and Information Sciences 2. Department of Primary and Community Care 3. Department of Pulmonary Diseases
Iwaya et al. [25]	Review	International Journal of Medical Informatics	Clinical medicine	Q1	2.716	12	1.Department of Computer and Digital Systems Engineering 2. Heart Institute (InCor) 3. Ericsson Research
Lee et al. [26]	Descriptive	International Journal of Medical Informatics	Clinical medicine	Q1	2.716	6	1. Department of Rheumatology and Immunology 2. Department of Clinical Research 3. Integrated Health Information Systems 4. Duke-NUS Graduate Medical School 5. Yong Loo Lin School of Medicine
Labrique et al. [27]	Expert opinion	International Journal of Medical Informatics	Clinical medicine	Q1	2.716	15	1. Johns Hopkins Bloomberg School of Public Health 2.Johns Hopkins School of Medicine 3. World Health Organization
Alnanih et al. [27]	Technological improvement	Procedia Computer Science	Not indexed	Not indexed	–	1	1. Department of Computer Science & Software Engineering 2. Department of Computer Science
Meneze et al. [29]	Technological development	Procedia Technology	Not indexed	Not indexed	–	2	1. S@BER: Tecnologias Educaionais e Sociais Research Group 2. Programa de Pós-Graduaçao em Engenharia Biomédica
Liu et al. [12]	Descriptive	Journal of Systems and Software	Computer science	Q2	1.245	35	1. School of EECS 2. Psychology Department
Datta et al. [45]	Technological development	Procedia Computer Science	Not indexed	Not indexed	–	4	1. NU Community Research Institute 2. School of Engineering & Technology 3.Illinois Department of Human Services
Surka et al. [46]	Clinical Trial	International Journal of Medical Informatics	Clinical medicine	Q1	2.716	4	1. Chronic Disease Initiative for Africa 2. Center for Online Health 3. Division of Cardiovascular Medicine 4. School of Public Health 5. Chronic Disease Initiative for Africa, Division of Diabetes and Endocrinology, Department of Medicine
Cornelius and Kotz [47]	Technological improvement	Pervasive and Mobile Computing	Computer science	Q2	1.667	3	1. Department of Computer Science 2. Institute for Security, Technology, and Society
Hao et al. [35]	Qualitative research	Computer Methods and Programs in Biomedicine	Computer science	Q2	1.093	0	1. Department of Cardiovascular Medicine 2. School of Health Care Administration 3. Graduate Institute of Biomedical Informatics 4. Institute of Biomedical Informatics 5. Office of Research and Development 6. Department of Dermatology
Balsam et al. [32]	Technological improvement	Biosensors and Bioelectronics	Chemistry	Q1	6.451	2	1. Division of Biology, Office of Science and Engineering, FDA 2. University of Maryland 3. Western Regional Research Center 4. Division of Cancer Biology, National Cancer Institute
Sezgin and Ozkan Yildirim [30]	Review	Procedia Technology	Not indexed	Not indexed	–	0	1. Middle East Technical University, Informatics Institute
Turner-McGrievya and Tateb [48]	Clinical Trial	International Journal of Medical Informatics	Clinical medicine	Q1	2.716	1	1. Department of Health Promotion, Education, and Behavior 2. Departments of Nutrition and Health Behavior
Wang et al. [15]	Descriptive	Journal Of Medical Internet Research mHealth and uHealth	Not indexed	Not indexed	–	2	1. Gerontechnology Lab 2. Environment and Health Group 3. Department of Psychiatry 4. Global Initiative on Care giving for the Elderly 5. College of Social Work 6. Department of Global Health and Social Medicine
Ploderer et al. [16]	Qualitative research	Journal Of Medical Internet Research mHealth and uHealth	Not indexed	Not indexed	–	1	1. Department of Computing and Information Systems 2. The Cancer Council Victoria
Kuo et al. [36]	Descriptive	Journal of the American Medical Informatics Association	Social science, general	Q1	3.932	0	1. Cathay General Hospital 2. Institute of Biomedical Informatics College of Nursing
Kizakevich et al. [49]	Technological development	Studies in Health Technology and Informatics (IOS Press)	Not indexed	Not indexed	–	2	1. RTI International, Research Triangle Park 2. National Center for PTSD, Veterans Health Affairs
Jibb et al. [40]	Technological development	Journal Of Medical Internet Research Research Protocols	Not indexed	Not indexed	–	4	1. Hospital for Sick Children 2. Lawrence S Bloomberg Faculty of Nursing 3. Institute of Health Policy 4. Center for Global eHealth Innovation
Sunyaev et al. [50]	Descriptive	Journal of the American Medical Informatics Association	Social science, general	Q1	3.932	2	1. Faculty of Management Economics and Social Sciences Department of Pediatrics, Boston Children´s Hospital, Harvard Medical School, The Petrie-Flom Center for Health Law Policy, Biotechnology, and Bioethics, Harvard Law School 2. Children´s Hospital Informatics Program at Harvard-MIT Health Sciences and Technology, Boston Children Hospital,Harvard Medical School
Lyons et al. [24]	Descriptive	Journal Of Medical Internet Research	Clinical medicine	Q1	4,669	3	1. Institute for Translational Sciences 2. Center for Interdisciplinary Research in Women´s Health 3. Department of Rehabilitation Sciences 4. College of Medicine 5. Department of Physical Therapy
Grindrod et al. [17]	Technological improvement	Journal Of Medical Internet Research mHealth and uHealth	Not indexed	Not indexed	–	0	1. School of Pharmacy 2. School of Public Health and Health Systems 3. Department of Family Medicine 4. Communitech 5. School of Optometry
Hundert et al. [14]	Descriptive	Journal Of Medical Internet Research mHealth and uHealth	Not indexed	Not indexed	–	5	1. IWK Health Center 2. Department of Community Health and Epidemiology 3. Departments of Pediatrics and Psychiatry 4. Hospital for Sick Children 5. Lawrence S. Bloomberg Faculty of Nursing
Martínez-Pérez et al. [51]	Review	Journal Of Medical Internet Research mHealth and uHealth	Not indexed	Not indexed	–	6	1. Department of Signal Theory and Communications and Telematics Engineering
Turner-McGrievy et al. [37]	Quasi-Experimental	Journal of the American Medical Informatics Association	Social science, general	Q1	3.932	24	1. Department of Health Promotion, Education, and Behavior 2. Department of Exercise Science 3. Department of Epidemiology and Biostatistics 4. Department of Nutrition and Department of Health Behavior
Carter et al. [52]	Descriptive	Annals of Vascular Surgery	Clinical medicine	Q3	1.029	8	1. Department of Clinical Surgery 2. Department of Clinical and Surgical Sciences (Surgery)
Cafazzo et al. [13]	Quasi-experimental	Journal Of Medical Internet Research	Clinical medicine	Q1	4.669	48	1. Center for Global eHealth Innovation 2. Institute of Health Policy 3. Institute of Biomaterials and Biomedical Engineering 4. Division of Adolescent Medicine 5. Department of Pediatrics 6. Division of Endocrinology
Abel et al. [53]	Technological development	Journal Of Medical Internet Research mHealth and uHealth	Not indexed	Not indexed	–	1	1. King´s College London, Institute of Psychiatry Department of Clinical Neuroscience, 2. Department of Pharmacology and Clinical Neuroscience 3. King´s College London, Department of Neuroscience
Mann et al. [23]	Quasi-Experimental	Studies in Health Technology and Informatics (IOS press)	Not indexed	Not indexed	–	1	1. Department of Medicine 2. Department of Computer Science 3. Department of Pediatrics
Chen et al. [54]	Protocol development	BMC Public Health	Social science, general	Q2	2.321	1	1. Department of Integrated Early Childhood Development 1. Global eHealth Unit, Department of Primary Care and Public Health 3. Save the Children China 4. Save the Children China Program 5. Save the Children
King et al. [38]	Descriptive	Global Health Action	Social science, general	Q2	1.646	0	1. Institute for Global Health 2. MaiMawna Project 3. Department of Computer Science 4. Baylor College of Medicine Children´s Foundation
van Drongelen et al. [33]	Clinical Trial	Scandinavian Journal of Work, Environment & Health	Social science, general	Q1	3.095	3	1. Department of Public and Occupational Health, EMGO Institute for Health and Care Research 2. KLM Health Services 3. Body@Work TNO VUmc, Research Center on Physical Activity Department of Clinical Epidemiology and Biostatistics
Bierbrier et al. [55]	Descriptive	Journal Of Medical Internet Research	Clinical medicine	Q1	4.669	10	1. University Health Network, Center for Innovation in Complex Care 2. University Health Network, Department of General Internal Medicine
O’Malley et al. [18]	Descriptive	Journal Of Medical Internet Research mHealth and uHealth	Not indexed	Not indexed	–	2	1. Department of Physiotherapy 2. Department of Epidemiology and Public Health 3. Child Health Information Center 4. School of Health Sciences 5. Department of Applied Psychology
Brooke and Thompson [56]	Expert opinion	Journal of diabetes science and technology	Not indexed	Not indexed	–	2	1. Vasoptic Medical 2. Epstein Becker Green
Dunford et al. [19]	Technological development	Journal Of Medical Internet Research mHealth and uHealth	Not indexed	Not indexed	–	3	1. The George Institute for Global Health 2. University of Sydney 3. Xyris Software 4. Bupa Australia 5. Key to nutrition
Masters [57]	Descriptive	Medical Teacher	Clinical medicine	Q1	2.045	1	1. Medical Education and Informatics Unit
Pulman et al. [20]	Qualitative research	Journal Of Medical Internet Research mHealth and uHealth	Not indexed	Not indexed	–	1	1. The School of Health & Social Care 2. Bournemouth University 3. Faculty of Health and Social Care 4. Diabetes Center
Parmanto et al. [58]	Descriptive	Journal Of Medical Internet Research mHealth and uHealth	Not indexed	Not indexed	–	2	1. Department of Health Information Management 2. Department of Rehabilitation Science & Technology 3. Department of Psychical Medicine & Rehabilitation
Vriend et al. [59]	Descriptive	British Journal Of Sports Medicine	Clinical medicine	Q1	4.171	0	1. VeiligheidNL 2. Department of Public and Occupational Health EMGO Institute for Health and Care Research
Pérez-Cruzado and Cuesta-Vargas [60]	Protocol development	BMC Public Health	Social science, general	Q2	2.321	0	1. Department of Physiotherapy 2. School of Clinical Science
Ribu et al. [41]	Protocol development	Journal Of Medical Internet Research Research Protocols	Not indexed	Not indexed	–	2	1. Department of Nursing 2. Department of Health Sciences 3. Norwegian Center for Integrated Care and Telemedicine 4. Institute of Clinical Medicine
Fiordelli et al. [61]	Review	Journal Of Medical Internet Research	Clinical medicine	Q1	4.669	37	1. Institute of Communication and Health, Faculty of Communication Sciences
Becker et al. [21]	Expert opinion	Journal Of Medical Internet Research mHealth and uHealth	Not indexed	Not indexed	–	8	1. Institute of Drug Safety, Department of Nephrology 2. Marketing Department 3. Lifepatch GmbH 4. Black Tusk AG 5. Division of Nephrology, University of Maryland School of Medicine 6. PL Reichertz Institute for Medical Informatics
Lewis and Wyatt [62]	Expert opinion	Journal Of Medical Internet Research	Clinical medicine	Q1	4.669	8	1. Warwick Medical School 2. Leeds Institute of Health Sciences, Faculty of Medicine, Health & Psychology
de la Vega and Miró [63]	Review	PLoS One	Multidisciplinary	Q1	3.534	3	1. Unit for the study and Treatment of Pain – ALGOS, Research Center for Behavior Assessment, Department of Psychology and Institute d´Investigació Sanitària Pere Virgili
Shishido et al. [64]	Descriptive	Studies in Health Technology and Informatics	Not indexed	Not indexed	–	0	1. Department of Compute Engineering 2. Collegiate Nursing Technician Course
Slaper and Conkol [65]	Expert Opinion	Pediatric Annals	Clinical medicine	Q4	0.306	0	1. Telemedicine Program Coordinator, Nationwide Children´s Hospital 2. Care Coordination Program Manager, Complex Care and Telehealth Nurse Clinician, Nationwide Children´s Hospital
Martínez-Pérez et al. [66]	Review	Journal Of Medical Internet Research mHealth and uHealth	Not indexed	Not indexed	–	9	1. Department of Signal Theory and Communications 2. Institute of Biomedical Engineering and Health Technology
Martínez-Pérez et al. [67]	Review	Journal of Medical Systems	Clinical medicine	Q3	1.372	20	1. Department of Signal Theory and Communications and Telematics Engineering 2. Biomedical Informatics Group, Instituto de Aplicaciones de las Tecnologías de la Información y de las Comunicaciones Avanzadas (ITACA)
Martínez-Pérez et al. [68]	Review	Journal Of Medical Internet Research	Clinical medicine	Q1	4.669	29	1. Department of Signal Theory and Communications and Telematics Engineering
Yang and Silverman [69]	Expert opinion	Health Affairs (Millwood)	Social science, general	Q1	4.321	13	1. Department of Health Policy and Management 2. Health and Policy Management & Public Health and Law
Boulos et al. [70]	Expert opinion	Online Journal of Public Health Informatics	Not indexed	Not indexed	–	9	1. Faculty of Health & Human Sciences 2. Dermatology Residency Program 3. Columbia Residency College of Physicians and Surgeons Klein Buendel Inc 4. Dermatology Service
Ahtinen et al. [71]	Quasi-experimental	Journal Of Medical Internet Research mHealth and uHealth	Not indexed	Not indexed	–	2	1. VTT Technical Research Center of Finland 2. Department of Psychology
Eskenazi et al. [34]	Technological development	Environment International	Environment, ecology	Q1	5.664	1	1. Center for Environmental Research and Children´s Health (CERCH), School of Public Health 2. Malaria Control Programme, Limpopo Department of Health 3. Malaria Control Programme, National Department of Health 4. Center for Sustainable Malaria Control, Department of Urology 5. Center for Information Technology in the Interest Society (CITRIS) Health Care Initiative 6. Environmental & Occupational Health Sciences Department
Barwais et al. [72]	Clinical Trial	Health and Quality of Life Outcomes	Clinical medicine	Q2	2.099	4	1. School of Exercise and Nutrition Sciences 2. Department of Physical Education and Sports 3. School of Education and Professional Studies
Tsui et al. [73]	Quasi-Experimental	Journal of Diabetes Science and Technology	Not indexed	Not indexed	–	0	1. Department of Ophthalmology 2. Department of Medicine and the Gonda Diabetes Center 3. Departments of Psychology and Psychiatry/Biobehavioral Sciences 4. Department of Ophthalmology and Jules Stein Eye Institute
Goldenberg et al. [74]	Qualitative research	Journal Of Medical Internet Research mHealth and uHealth	Not indexed	Not indexed	–	1	1. Department of Epidemiology 2. Department of Medicine, Division of Allergy and Infectious Diseases 3. Hubert Department of Global Health
Bricker et al. [39]	Clinical Trial	Drug and Alcohol Dependence	Neuroscience & behavior	Q1 & Q2	3.278	9	1. Fred Hutchinson Cancer Research Center, Division of Public Health Sciences 2. Department of Psychology 3. Department of Human Centered Design and Engineering 4. Department of Psychiatry and Behavioral Sciences
Lopez et al. [22]	Technological development	Journal Of Medical Internet Research mHealth and uHealth	Not indexed	Not indexed	–	0	1. Center for Innovation and Health Education 2. School of Medicine and Health Sciences 3. Systems and Computing Engineering Department 4. School of Medicine
Mobasheri et al. [75]	Review	Breast	Clinical medicine	Q3 & Q1	2.581	0	1. Department of Surgery & Cancer 2. Institute of Global Health Innovation 3. Department of Breast Surgery
Zmily et al. [76]	Quasi-experimental study	Journal Of Medical Internet Research mHealth and uHealth	Not indexed	Not indexed	–	0	1. School of Computer Engineering and Information Technology 2. Darat Samir Shamma
Mirkovic et al. [77]	Technological development	Journal Of Medical Internet Research mHealth and uHealth	Not indexed	Not indexed	–	3	1. Center for Shared Decision Making and Collaborative Care Research 2. Department of Biomedical Informatics
Klonoff [78]	Review	Journal of Diabetes Science and Technology	Not indexed	Not indexed	–	6	1. Mills-Peninsula Health Services
van der Weegen et al. [79]	Technological development	Journal Of Medical Internet Research mHealth and uHealth	Not indexed	Not indexed	–	11	1. CAPHRI School for Public and Primary Care, Department of Health Services Research 2. Research Center Technology in Care 3. CAPHRI School for Public Health and Primary Care, Department of General Practice
Leal Neto et al. [80]	Technological development	Journal Of Medical Internet Research mHealth and uHealth	Not indexed	Not indexed	–	0	1. Aggeu Magalhaes Research Center 2. PPGIA, Department of Statistics and Informatics
Albrecht et al. [81]	Descriptive	Journal Of Medical Internet Research mHealth and uHealth	Not indexed	Not indexed	–	1	1. P.L. Reichertz Institute for Medical Informatics 2. Nursing Department
Hilliard et al. [82]	Qualitative research	Journal Of Medical Internet Research mHealth and uHealth	Not indexed	Not indexed	–	2	1. Baylor College of Medicine, Department of Pediatrics 2. Nationwide Children´s Hospital 3. Johns Hopkins University School of Medicine, Department of Medicine
Arnhold et al. [83]	Descriptive	Journal Of Medical Internet Research	Clinical medicine	Q1	4.669	11	1. Research Association Public Health Saxony and Saxony-Anhalt
Breton et al. [84]	Descriptive	Translational Behavioral Medicine: Practice, Policy, Research	Not indexed	Not indexed	–	42	1. The George Washington University of Public Health and Health Services 2. Deparment of Community and Family Medicine and 3. Psychology and Neuroscience
BinDhim et al. [85]	Descriptive	Journal Of Medical Internet Research mHealth and uHealth	Not indexed	Not indexed	–	2	1. Sydney Medical School, Department of Public Health 2. Public Health and Health Informatics School
Breland et al. [86]	Descriptive	Translational Behavioral Medicine: Practice, Policy, Research	Not indexed	Not indexed	–	5	1. Department of Psychology 2. Institute for Health, Health Care Policy and Aging Research
Silva et al. [87]	Technological development	Journal Of Medical Internet Research	Clinical medicine	Q1	4.669	7	1. Instituto de Telecomunicaçoes 2. Nanjing University of Posts and Telecomunications
Mann et al. [88]	Technological development	Journal Of Medical Internet Research mHealth and uHealth	Not indexed	Not indexed	–	1	1. Department of Medicine 2. Department of General Internal Medicine 3. Department of Preventive Medicine & Epidemiology 4. Deparment of Computer Science
Aguilera et al. [89]	Technological development	Journal of Affective Disorders	Psychiatry, psychology	Q2	3.383	1	1. University of California, Berkeley, US 2. Northwestern University, US 3. University of California, San Francisco, US
Almunawar et al. [90]	Review	Health Policy and Technology	Not indexed	Not indexed	–	0	1. School of Business & Economics, Universiti Brunei 2. CEC & Joint Appointment e-Government Innovation Centre, Universiti Brunei 3. Department of Health Policy & Management, Jackson State University, US
Anwar et al. [91]	Expert Opinion	Health Policy and Technology	Not indexed	Not indexed	–	0	1. Department of Computer Science, North Carolina A&T State University, USA 2. School of Information Sciences, University of Pittsburgh, USA 3. Division of Information Systems, McMaster University, Canada
Azzazyand Elbehery [92]	Review	Clinica Chimica Acta	Clinical medicine	Q1	2.824	0	1. Novel Diagnostics and Therapeutics, Yousef Jameel Science & Technology Research Centre, and Department of Chemistry, School of Sciences & Engineering, The American University in Cairo,Egypt 2. Graduate Program of Biotechnology, School of Sciences and Engineering, The American University in Cairo, Egypt
Boissin et al. [93]	Descriptive	Burns	Clinical medicine	Q4	1.880	0	1. Department of Public Health Sciences, Karolinska Institutet, Sweden 2. Stellenbosch Institute for Advanced Study (STIAS), Wallenberg Research Centre at Stellenbosch University,, South Africa 3. University of South Africa, Preller Street, Pretoria, South Africa 4. Division of Emergency Medicine, Faculty of Medicine and Health Sciences, Stellenbosch University, South Africa
Bradway et al. [94]	Expert opinion	Trends in Endocrinology and Metabolism	Social science, general	Q1	9.392	0	1. Norwegian Centre for Integrated Care and Telemedicine (NST), University Hospital of North Norway, Norway 2. US Department of State Bureau of Educational and Cultural Affairs and IIE: United States of America- Norway Fulbright Program,, Norway
Chang et al. [95]	Technological development	Computer Methods and Programs n Biomedicine	Computer science	Q1	1.897	0	1. Department of Engineering Science, National Cheng Kung University,Taiwan 2. Department of Nursing, College of Medicine, National Cheng Kung University, Taiwan 3. Department of Medical Informatics, National Cheng Kung University Hospital, College of Medicine, Taiwan
Danaher et al. [96]	Review	Internet Interventions	Not indexed	Not indexed	–	3	1. Oregon Research Institute, Eugene, USA 2. Norwegian Centre for Addiction Research, University of Oslo, Norway 3. IEQ Technology, Springfield, USA
Green et al. [97]	Expert Opinion	Journal of the American Society of Hypertension	Clinical medicine	Q3	2.606	0	1. Group Health Cooperative and Group Health Research Institute, Seattle, WA, USA
Guo et al. [98]	Technological development	Computers in Industry	Computer science	Q3	1.287	0	1. Department of Nursing, Mackay Medical College, New Taipei, Taiwan 2. Department of Information Management, Chang Gung University, Taoyuan, Taiwan
Helf and Hlavacs [99]	Review	Entertainment Computing	Not indexed	Not indexed	–	0	1. University of Vienna, Research Group Entertainment Computing, Faculty of Computer Science, Währinger Straße 29, 1090 Vienna, Austria
Jain et al. [100]	Descriptive	Asian Journal of Psychiatry	Not indexed	Not indexed	–	0	1. Department of Psychiatry, Pt. B.D.S. Postgraduate Institute of Medical Sciences, Rohtak, Haryana, India 2. Department of Psychiatry, SP Medical College, Bikaner, Rajasthan, India 3. Department of Psychiatry, Dr. SN Medical College, Jodhpur, Rajasthan, India 4. Department of Psychiatry, SMS Medical College, Jaipur, Rajasthan, India
Kramer et al. [101]	Expert Opinion	Cognitive and Behavioral Practice	Psychiatry, psychology	Q3	1.562	1	1. National Center for Telehealth and Technology. Joint Base Lewis McChord, USA
Kumar et al. [102]	Descriptive	Journal of the American Society of Hypertension	Clinical medicine	Q3	2.606	1	1. Department of Medicine, Cambridge Health Alliance, Harvard Medical School, USA; 2. Department of Medicine, All-India Institute of Medical Sciences, New Delhi, India; 3. Department of Medicine, University of Texas Southwestern Med Center, USA 4. Division of Nephrology, Department of Medicine, Beth Israel Deaconess Medical Center, Harvard Medical School, Boston, USA
Lucivero and Prainsack [103]	Expert Opinion	Applied and Translational Genomics	Not indexed	Not indexed	–	0	1, Social Science Health and Medicine Department, King’s College London, United Kingdom
Maciel and Hayashi [104]	Technological development	Procedia Manufacturing	Not indexed	Not indexed	–	0	1. Universidade Federal de Pernambuco,Brazil 2. UniversidadeFederal do Amazonas, Brazil
McCarroll et al. [105]	Clinical Trial	Gynecologic Oncology	Clinical medicine	Q1	3.774	2	1. Summa Health System, Akron, OH, USA 2. Youngstown State University, Youngstown, OH, USA 3. Northeast Ohio Medical University (NEOMED), USA
Nocum et al. [106]	Technological development	Procedia Manufacturing	Not indexed	Not indexed	–	0	1. BS Industrial Engineering student, Department of Industrial Engineering and Operations Research, UP Diliman, Philippines 2. Instructor, Department of Industrial Engineering and Operations Research, UP Diliman, 1101, Philippines
Nunes and Simões-Marques [107]	Descriptive	Procedia Manufacturing	Not indexed	Not indexed	–	0	1. Faculdade de Ciências e Tecnologia, Universidade Nova de Lisboa 2. UNIDEMI, Campus de Caparica, Caparica, Portugal 3. CINAV-Portuguese Navy, Alfeite, ALMADA, Portugal
Olla et al. [108].	Technological development	Health Policy and Technology	Not indexed	Not indexed	–	0	1. Madonna University, Livonia 48150, USA 2. McMaster University, West Hamilton, Canada 3.iSTOC Automaatiotie 1, 90460 Oulunsalo, Finland
Ovbiagele [109]	Review	Journal of Stroke and Cerebrovascular Diseases	Neuroscience & behavior	Q4	1.669	0	1. Department of Neurology and Neurosurgery, Medical University of South Carolina, Charleston, South Carolina.
Paschou et al. [110]	Technological development	The Journal of Systems and Software	Computer science	Q2	1.352	0	1. Department of Computer Engineering & Informatics, School of Engineering, University of Patras,Greece 2. DFG-Center for Regenerative Therapies Dresden (CRTD), TU Dresden, Germany
Patterson et al. [111]	Qualitative research	Seizure	Neuroscience & behavior	Q3	1.822	0	1. Dhulikhel Hospital, Kavre, Nepal 2. All India Institute of Medical Sciences, New Delhi, India 3. Kathmandu Model Hospital, Kathmandu, Nepal 4. All India Institute of Medical Sciences, New Delhi, India
Schnall and Iribarren [112]	Review	American Journal of Infection Control	Inmunology	Q2	2.206	0	1. Columbia University School of Nursing, New York
Silva et al. [113]	Review	Journal of Biomedical Informatics	Computer science	Q1	2.126	0	1. Instituto de Telecomunicações, University of Beira Interior, Covilhã, Portugal 2. Center of Excellence in Information Assurance (CoEIA), King Saud University, Saudi Arabia 3. Department of Signal Theory and Communications, University of Valladolid, Valladolid, Spain
Sindi et al. [114]	Technological development	Alzheimer’s & Dementia: Diagnosis, Assessment & Disease Monitoring	Not indexed	Not indexed	–	0	1. Aging Research Center (ARC), Department of Neurobiology, Care sciences and Society (NVS), Karolinska Institutet, Stockholm, Sweden 2. Karolinska Institutet Center for Alzheimer Research, Department of Neurobiology, Care sciences and Society (NVS), Stockholm, Sweden 3. Merz Pharmaceuticals GmbH, Frankfurt am Main, Germany 4. Department of Chronic Disease Prevention, National Institute for Health and Welfare, Helsinki, Finland 5. Department of Neurology, Institute of Clinical Medicine, University of Eastern Finland, Finland 6. Neurocenter, Department of Neurology, Kuopio University Hospital, Finland 7. Centre for Vascular Prevention, Department for Clinical Neurosciences and Preventive Medicine, Danube-University Krems, Austria 8. Diabetes Research Group, King Abdulaziz University, Jeddah, Saudi Arabia
M.J. hompson and Valdez [115]	Descriptive	Health Policy and Technology	Not indexed	Not indexed	–	0	1. Department of Public Health Sciences, University of Virginia, United States
Waldman and Stevens [116]	Qualitative research	Reproductive Health Matters	Not indexed	Not indexed	–	0	1. Institute of Development Studies, University of Sussex, UK. 2. WISH Associates and African Gender Institute, University of Cape Town
Yang et al. [117]	Qualitative research	Preventive Medicine Reports	Not indexed	Not indexed	–	0	1. Department of Kinesiology, The Pennsylvania State University, USA Department of Preventive Medicine, Northwestern University Feinberg School of Medicine, Chicago, USA

**Table 2:** Main results and conclusions of reviewed papers

Paper	Author/year	Main results and conclusions
Paper 1: Assessment of the health IT usability evaluation model (Health-ITUEM) for evaluating mobile health (mHealth) technology	Brown et al. [43]	This work analyzes the assessment model ITUEM (Health-ITUEM) usability in adolescents when evaluating the usability of Health Apps
Paper 2: Capillary array waveguide amplified fluorescence detector for mHealth	Balsam et al. [44]	This work aims to improve the operation of the optical detector in mobile phones through the expansion of fluorescent signals to be used in health apps with diagnostic purposes
Paper 3: Development and validation of an instrument to Measure user perceived service quality of mHealth	Akter et al. [31]	Pilot study to develop a scale for measuring the quality perceived by the user regarding the mHealth which showed a clear link between service quality and satisfaction, as well as satisfaction and continuity in use, as well as between the quality of service and continued use
Paper 4: An autonomous mobile system for the management of COPD	Van der Heijden et al. [25]	Work based on the development of an app to detect exacerbations in COPD patients through the use of a spirometer and pulse oximeter. The evaluation showed that the model can reliably detect exacerbations and the pilot study suggests that an intervention based on this system may be successful
Paper 5: Mobile health in emerging countries: A survey of research initiatives in Brazil	Iwaya et al. [25]	Systematic review to see the state of development of mHealth initiatives in Brazil. Most projects were focused on health surveys and surveillance and patient records and monitoring, the majority being deployed as prototypes for testing and being supported by the university
Paper 6: The feasibility of using SMS as a health survey tool: An exploratory study in patients with rheumatoid arthritis	Lee et al. [26]	This research tests the feasibility of using SMS as a tool for the study of patients with rheumatoid arthritis. It proved feasible, reducing response time and at a lower price than mail or postal mail
Paper 7: H_pe for mHealth: More “y” or “o” on the horizon?	Labrique et al. [27]	Letter to the editor in which appear a criticism of the changes and development that is occurring in mHealth, with a saturation of pilot studies, unclassifiable designs impossible to extrapolate to larger sample sizes and with a lack of evidence
Paper 8: Context-based and rule-based adaptation of mobile user interfaces in mHealth	Alnanih et al. [28]	Study where a user interface (MUI) is developed to serve as a bridge between any application and the health professional. The results showed that the proposed improvement did not increase the efficacy, safety, navigation, productivity or efficiency in the work. Neither it increased its satisfaction
Paper 9: A Proposal of mobile system to support scenario-based learning for health promotion	Menezes et al. [29]	The document presents “KNOW Communities” which includes a series of virtual scenarios where the goal is to present health problems for students, workers and population, allowing developing educational activities. No results are displayed
Paper 10: Status and trends of mobile-health applications for iOS devices: A developer’s perspective	Liu et al. [13]	Study of 159 applications from the AppStore, approached from the perspective of developers, looking at features like architecture, interface design, etc. The purpose of the text is to be used as a reference and guide for anyone who wants to develop on iOS
Paper 11: mCHOIS: An Application of mobile technology for childhood obesity surveillance	Datta et al. [45]	The development of an application is described to facilitate the collection of data (mChois) that aims to overcome the limitations of a web for childhood obesity (chois) that depended on the Internet. The application allows the collection and storage of data locally and is now being used by “The Illinois Department of Human Services (IDHS)” for school health program
Paper 12: Evaluating the use of mobile phone technology to enhance cardiovascular disease screening by community health workers	Surka et al. [46]	The purpose of this study is to develop an application that assess the risk of cardiovascular disease, assessing its impact on health staff and the duration of the screening, compared with paper-based. The application was considered easy to use, faster and more accurate, but with a worse visual display to explain the risk to the population
Paper 13: Recognizing whether sensors are on the same body	Cornelius and Kotz [47]	The proposition of this paper is to improve connectivity with sensors Smartphone. It shows a probabilistic model to look for correlations between sensors and accelerometers of your device to avoid the above mentioned problem. The new model achieved an accuracy of 85 %
Paper 14: LabPush: a pilot study of providing remote Clinics with laboratory results via short message service (SMS) in Swaziland, Africa—a qualitative study	Hao et al. [35]	The study presents the use of short message service (SMS) “LabPush” to send the most important results to the professionals, to facilitate and accelerate decision-making. SMS method shortened the turnaround time of results. The professionals expressed satisfaction at the prospect of starting treatment earlier, because communication with the laboratory was improved and if results were lost, it was easily recovered by a call
Paper 15: Thousand-fold fluorescent signal Amplification for mHealth diagnostics	Balsam et al. [32]	Capillary array focus and image stacking computer that is capable of amplifying the weak fluorescent signals, thereby improving the sensitivity of the optical sensors of mobile devices. It is the same studio as Article 2
Paper 16: A literature review on attitudes of health professionals towards health information systems: from e-Health to m- Health	Sezgin and Ozkan Yildirim [30]	The study presents a review of the acceptance of health information systems by health professionals. Through various theoretical models it gets explained the purpose of the health professionals in the use of health technologies with high rates of variability
Paper 17: Are we sure that Mobile Health is really mobile? An examination of mobile device use during two remotely- delivered weight loss interventions	Turner-McGrievya and Tateb [37]	This study compared the effectiveness in terms of access to information in the traditional way (desktops, laptops…) against mobile, and if they affect the results of commitment and health in a weight loss intervention in 137 patients. It showed greater weight loss for those who used the mobile device
Paper 18: A Classification scheme for analyzing mobile apps used to prevent and manage disease in late life	Wang et al. [15]	The study carried out the classification of 119 mobile applications from the AppStore in the categories of health and welfare for the elderly. The classification was carried out satisfactorily, reaching 100 % agreement between what was developed in the study and what was conducted by two external encoders. This system give you a view of the distribution of applications to developers and allow users to know which one suits your needs.
Paper 19: A data encryption solution for mobile health apps in cooperation environments	Silva et al. [87]	The study has the goal to develop a system of data encryption (DE4MHA) to guarantee the security of data. That the app had a similar behaviour to the one it would have without the data encryption system was achieved, so that quality was also guaranteed. The system could be adapted to other apps of mHealth
Paper 20: Adherence to evidence-based guidelines among diabetes self-management apps	Breland et al. [86]	The research has the goal to evaluate if 411 apps used for diabetes self-management followed the guidelines based on scientific evidence. From the 7 self- management behaviours recommended by the American Association of Diabetes Educators (AADE) most of them just accomplished 1 or 2
Paper 21: A mobile app offering distractions and tips to cope with cigarette craving: a qualitative study	Ploderer et al. [16]	The article shows the development of the app “DistractMe” which permits whoever wants to stop smoking, the access to different type of distractions and tips to cope with smoking craving. With a small simple of 14 smokers, 5 patients reported a favourable use of the evasive technique and other 9 reported a favourable use permiting to avoid smoking instead of other activities
Paper 22: A newborn baby care support app and system for mHealth	Kuo et al. [36]	The study describes the development of an app with two subsystems “Baby’s Health Record System (BHRS) and “Baby Care Consultation System” (BCCS) which allows mums to have a better care of their newborn. From the 64 women studied, the most of them were satisfied with the app
Paper 23: A Personal health information toolkit for health intervention research	Kizakevich et al. [49]	The article presents the development of the toolkit PHIT(Personal Health Intervention Toolkit) to facilitate the investigation and development of applications in health interventions in chronic diseases, health control with data collection, etc. Most of the 31 patients who participated, valued the app over percentile 95.
Paper 24: A smartphone-based pain management app for adolescents with cancer: establishing system requirements and a pain care algorithm based on literature review, interviews, and consensus.	Jibb et al. [40]	The development of an algorithm of decision was aimed in base on the evidence for the better pain care in adolescents with cancer. It was determined that the interventions including the support of therapists are more efficient and it was recommended to include patients and/or relatives both in the development and in the design of the app
Paper 25: Availability and quality of mobile health app privacy policies	Sunyaev et al. [50]	The document presents an analysis in the privacy policy of the apps of mHealth.The analysis is focused on the 600 mobile Health app more used for Android and IOS. It showed that only the 30 % had privacy policy and these were insufficient
Paper 26: Behavior change techniques implemented in electronic lifestyle activity monitors: a systematic content analysis	Lyons et al. [24]	The study analyses 13 electronic lifestyle activity monitors to find out which of the fourteen techniques identified in the literature are founded with the aim of increasing them. All them had a similar behaviour, suing the app to direct the information. The techniques and recommendations were based on the scientific literature. From the 14 identified as potentially efficient in behaviour change, the monitors included between 5 and 10
Paper 27: ClereMed: lessons learned from a pilot study of a mobile screening tool to identify and support adults who have difficulty with medication labels	Grindrod et al. [17]	The study presents a prototype of an app (“ClereMed”) to identify people who have difficulty reading, poor ability in handling medication and cognitive impairment, and to assess the acceptance of touch devices in older people. We Studied 47 elderly. 84 % of participants stated that the application was easy to use. In terms of reading difficulty, it was correctly identified 72 %.Only 21 % of participants showed cognitive difficulties
Paper 28: Commercially available mobile Pone headache diary apps: a systematic review	Hundert A.S., et al. [14]	In this paper 7 rules guideline was created as minimum requirements for considering that an application was useful in the management and control of headache. 38 apps were studied. None of them met all the criteria and neither was published in scientific literature, and only 3 met five of them. It showed a lack of scientific knowledge and evidence in this type of application
Paper 29: Comparison of mobile apps for the leading causes of death among different income zones: a review of the literature and app stores	Martínez-Pérez et al. [51]	It is a review that seeks to compare research and the number of mobile applications for diseases and conditions that are major causes of death according to the WHO, based on the income of regions. The results show applications of high-income regions are more common, with some exceptions such as HIV/AIDS and cancer of airway. It shows more effort in commercial work than in the research in this application field
Paper 30: Comparison of traditional versus mobile app self- monitoring of physical activity and dietary intake among overweight adults participating in an mHealth weight loss program	Turner-McGrievy et al. [37]	The paper presents a study of 96 overweight individuals, whose purpose is to analyse the relationship between diet, self-monitoring of physical activity and eating habits by following different types of monitoring (mobile applications, web diary on paper). The group using the mobile application to control physical activity showed better results
Paper 31: Contemporary vascular smartphone medical applications	Carter et al. [52]	The text presents a search for the availability of applications with vascular diseases as a theme in main application stores, as well as possible integration in practice. 49 were located, of which only 13 documented the participation of a medical professional in the design or content. It was concluded that the use of mHealth could be potentially beneficial, but it also showed a lack of high scientific quality apps
Paper 32: Design of an mHealth app for the self-management of adolescent type 1 diabetes: a pilot study	Cafazzo et al. [42]	The objective is the design, development and implementation of a study for the management of type 1 diabetes in 20 adolescents. There was an increase in daily average frequency of measurement by 50 %. Another study is needed to assess the improvement of HbA1c
Paper 33: Development of a Smartphone App for a Genetics Website: The Amyotrophic Lateral Sclerosis Online Genetics Database (ALSoD)	Abel et al. [53]	The paper presents the development of a mobile version of the web “Genetics The ALS Online Database (ALSoD)” and an application for smartphone which allows easier and wider access. It showed an increase of 26 % of visits to the website and a 230 % increase in visits through mobile devices
Paper 34: Development of DASH Mobile a mHealth lifestyle change intervention for the management of hypertension	Mann et al. [23]	It presents the development of “Mobile DASH” application for Android, based on the DASH diet for the control and management of hypertension is exposed. No results included
Paper 35: Effectiveness of a smart phone app on improving immunization of children in rural Sichuan Province, China: study protocol for a paired cluster randomized controlled trial	Chen et al. [54]	It presents the protocol of a study evaluating the effectiveness of an application to increase the level of immunization in the province of Sichuan (China) The primary outcome is the total dose coverage and the secondary is the coverage in other five vaccines of the expanded program of immunization. No results are shown
Paper 36: Electronic data capture in a rural African setting: evaluating experiences with different systems in Malawi	King et al. [38]	The text seeks to highlight the advantages and disadvantages of different systems of electronic data capture (EDC) used simultaneously in rural Malawi, to increase information and research. Workers preferred the EDC to paper-based systems and create a profitable system in the long term
Paper 37: Evaluation of an mHealth intervention aiming to improve health-related behavior and sleep and reduce fatigue among airline pilots	van Drongele et al. [33]	The study shows the effects of an intervention of mHealth in 502 airline pilots to reduce problems of fatigue, sleep and improve their perception of health through the app “MORE energy”. The intervention group improved quality of sleep, fatigue, strenuous physical activity, diet and rest
Paper 38: Evaluation of the accuracy of smartphone medical calculation apps	Bierbrier et al. [55]	The objective of this document is to analyze the accuracy of smartphone medical calculation apps offered by different stores 14 apps were analyzed and 13 functions for each of them. 10 cases for each one were carried out, being the results compared to the manual calculations. The total accuracy was 98,6 % and 43 % of the apps had an accuracy of 100 %
Paper 39: Exploring the usability of a mobile app for adolescent obesity management	O’Malley et al. [18]	The study proved the usability of the application “Reactivate” for the control of obesity in 10 adolescents. There were not showed results about the clinical effectiveness, although it was evaluated that it was more efficient It was also collected the need of the app working for iPhone, that the colours were brighter and the text bigger
Paper 40: Food and Drug Administration regulation of diabetes-related mHealth technologies	Brooke and Thompson [56]	The text tries to shed light on the regulation of FDA about the applications related qwith diabetes and it contributes with a convincing conclusion by which more clarity is required in the regulation of the applications devoted to diabetes
Paper 41: FoodSwitch: A Mobile Phone App to Enable Consumers to Make Healthier Food Choices and Crowdsourcing of National Food Composition Data	Dunford et al. [19]	The paper describes the development of a mobile application (“FootSwitch”) to help Australian population to a better understanding of the nutritional information of the foods in order to facilitate a better choice, using a database of 17,000 packaged foods that could be increased through “crowd-sourcing”
Paper 42: Health professionals as mobile content creators: teaching medical students to develop mHealth applications	Masters [57]	The research highlights the need to train medical students in the development of applications. “IBuildApp” is presented as applications development environment.107 students were surveyed about the perception of the project. The most perceived need was to increase learning, specially the programming which was considered the main factor of influence. The impact of the experience was similar to other studies
Paper 43: Ideas and enhancements related to mobile applications to support type 1 diabetes	Pulman et al. [20]	The objective of this study was to develop an application for the management of type 1 diabetes in young people. The app was aimed to offer quality services and improve the own “health-related quality of life (HRQOL)” of users, with input from experts and end-user over design. The researchers conducted the development of three prototypes of which one was chosen by nine respondents. Thus the data for prototyping and improving existing ones were taken, trying to suit the needs of the age group
Paper 44: iMHere: A novel mHealth system for supporting self-care in management of complex and chronic conditions	Parmanto et al. [58]	The paper presents the development of one mobile application titled “IMHere” and a medical portal to provide two-way communication. The application has the function of supporting self-care tasks, receiving and sending data of adherence and self-care regimes under supervision. The clinical phase of the study was being conducted. No results are shown
Paper 45: Implementation of an App-based neuromuscular training programme to prevent ankle sprains: a process evaluation using the RE-AIM Framework	Vriend et al. [59]	The paper presents an evaluation of the effectiveness of the App “Versterk je Enkel” focused on the prevention of recurrent ankle sprains. The application reached only 2.6 % (n = 82) of the target population. The use of the resource pointed that compliance with the integrated program was low. Efforts to ensure the proper collection and the use of app by the target population are needed
Paper 46: Improving adherence physical activity with a smartphone application based on adults with intellectual disabilities (APPCOID)	Pérez-Cruzado and Cuesta-Vargas [60]	Given the low levels of physical activity in people with mental disabilities, a research protocol aimed to improving the adherence to physical activity (based on the use of an app) in these patients is presented. No results are displayed
Paper 47: Low- intensity self-management intervention for persons with type 2 diabetes using a mobile phone-based diabetes diary, with and without health counseling and motivational interviewing: protocol for a randomized controlled trial	Ribu et al. [41]	The document presents a research aimed to assess the effectiveness in controlling diabetes of 151 patients, using three different interventions, one of them based on the use of one app. 17.2 % of the participants were lost to follow up at the publication time and 7.3 % were still in the trial. It indicated that the results would be published in 2014
Paper 48: Mapping mHealth Research: A Decade of Evolution	Fiordelli et al. [61]	The paper provides an overview of how the emergence of the new generation of phones has led to research on eHealth between 2002 and 2012, studying a sample of 117 papers. Most of them were published in medical journals, highlighting studies aimed to assess the control of chronic diseases through the use of mobile devices. It is concluded that mapping the evolution of this topic will allow a better understanding of their strengths and weaknesses, providing a very useful information for future developments
Paper 49: mHealth 2.0: experiences, possibilities, and perspectives	Becker et al. [21]	The paper is an opinion letter referring to the importance of mHealth as a tool to provide universal access to services, the importance of taking into account developmental psychosocial variables and the model of technological development of countries. It also says that criticism and reviews help users to trust these applications and test them, so inter-professional collaboration becomes essential in mHealth 2.0
Paper 50: mHealth and mobile medical Apps: a framework to assess risk and promote safer use	Lewis and Wyatt [62]	The document identifies various risks that mobile applications can pose to patient safety. A generic risk framework is also developed for users and developers, being a useful tool for the evaluation of specific applications in any particular context
Paper 51: mHealth: a strategic field without a solid scientific soul. a systematic review of pain-related apps	de la Vega and Miró [63]	A systematic review of the pain-related apps available in scientific databases and the main application shops was carried out to provide an overview of the current state of development of mHealth. 47 papers and 283 apps were found, showing a big gap between the research part and trade, since the vast majority of applications was not supported by scientific evidence
Paper 52: mHealth data collector: an application to collect and report indicators for assessment of cardiometabolic risk	Shishido et al. [64]	The paper presents “mHealth Data Collector”, an application that contains data and information associated with metabolic risk. After interviewing 45 health professionals who had used it, the researchers evidenced a reduction of time in the interview, a better collection, organization and retrieval of data, as well as standardization of the information entered into the system. The assessment of usability (ease of use, charging time, selecting the screen resolution, etc.) was also very positive
Paper 53: mHealth tools for the pediatric patient-centered medical home	Slaper and Conkol [65]	This paper is a review where performance and conceptual framework of some tools to perform the “pediatric patient-centered medical home (PCMH)” are discussed. This PMTCT is defined as a place to care for this type of patient which have to be accessible, continuous, comprehensive, family-centered, coordinated, compassionate and culturally effective. The document presented mHealth as a resource that can help to satisfy this necessity
Paper 54: Mobile applications for diabetics: a systematic review and expert-based usability evaluation considering the special requirements of diabetes patients age 50 years or older	Arnhold et al. [83]	This document is a systematic review of 656 applications available to assist in the self-management of diabetes mellitus types 1 and 2, considering the range of functions, groups of target users, etc. 10 % was used to evaluate the usability. An increase in the number of apps from 2008 to 2013 was appreciated and most ot them offered only one function and were intended for patients. 34.5 % provided general information about the disease. Usability decreased with increasing number of functions and was moderate to good for users over 50 years
Paper 55: Mobile apps in cardiology: review	Martínez-Pérez et al. [66]	The study presents a review of the literature of the main databases (406 papers) and applications stores (710 apps) to study the resources available with respect to cardiovascular disease. Only few items related to mobile applications were found. A great disparity was evident in the field of cardiology applications, having a lot of research on certain issues, while others were abandoned. It also emphasized that there was no link between the offered apps and the scientific production
Paper 56: Mobile clinical decision support systems and applications: a literature and commercial review	Martínez-Pérez et al. [67]	The document presents a review of applications developed to help in clinical decision making (CDDS) at the main databases (92 items) and the Android and Apple stores (192 apps). Articles made explicit that apps were developed for research purposes and were not available in stores. The Apps available were devoted to general practice and almost half directed to specialists. Developers focused on diseases that are the main cause of death, more prevalent and more disabling
Paper 57: Mobile health applications for the most prevalent conditions by the World Health Organization: review and analysis	Martínez-Pérez et al. [68]	Based on the eight most prevalent health conditions marked by WHO in 2004, the study presents a review of the literature (247 papers) in different databases and applications (3673 apps) of the main stores. Distribution of mobile applications did not correspond to the prevalence of health conditions. Results also found less applications in the literature, which led to think that there is more economic commercial motivation than research reasons. Diabetes and depression, health conditions prevalent in developed countries, were the focus of most research and applications
Paper 58: Mobile health applications: the patchwork of legal and liability issues suggests strategies to improve oversight	Yang and Silverman [69]	The text shows the state of the US legislation regarding mHealth. The lack of regulation regarding medical leaves, responsibility, possible negligence by the professional using an application, privacy when storing patient data, etc., are complex issues to cover despite legislative efforts
Paper 59: Mobile medical and health apps: state of the art, concerns, regulatory control and certification	Boulos et al. [70]	The document presents a review of studies evaluating health applications. The concept of “application as a medical device” and regulation that currently exists in the US and Europe is also discussed. An example of voluntary application for certification in the US market named “Happtique Health App Certification Program” is presented in the text. As to make a thorough control of the apps is very complicated, it is proposed to educate patients and users about what is the best use to give to these applications
Paper 60: Mobile mental wellness training for stress management: feasibility and design implications based on a one-month field study	Ahtinen et al. [71]	The paper is focused on the study of the use and results of the “Oiva” application, which was developed for the prevention and management of work-related stress and mental health problems in 15 workers. Significant changes were observed in the scores of stress and satisfaction with life, but not in the psychological flexibility. A significant increase was also evident in the improvement or maintenance of well-being, learning new skills and gain new ideas. It was perceived as easy to use, acceptable and useful
Paper 61: mSpray: a mobile phone technology to improve malaria control efforts and monitor human exposure to malaria control pesticides in Limpopo, South Africa	Eskenazi et al. [34]	The paper examines the effectiveness and usability of the app “mSpray” in 13 workers. The app was created to collect data from areas under sprays to control malaria, seeking to improve the currently used, paper-based system. The application included geo-location and use of spreadsheets to lead a real-time observation. The times were improved and errors with the above method were detected. Workers preferred “mSpray” system over the traditional system.
Paper 62: Physical activity, sedentary behavior and total wellness changes Among sedentary adults: a 4-week randomized controlled trial	Barwais et al. [72]	The study presents a four-week intervention program with 33 individuals using a staff monitor online activity, designed to reduce sedentary time and increasing physical activity. Results were compared to control group which continued normal life. Sedentary time was reduced in the intervention group, and the time of light, moderate and intense activity was increased. Researchers do not appreciate changes in the control group
Paper 63: Pilot study using mobile health to coordinate the diabetic patient, diabetologist, and ophthalmologist	Tsui et al. [73]	The paper presents a pilot study with 60 diabetic patients to assess the “Sightbook” application, which seeks the coordination between the patient, the physician who follows the diabetes patient and ophthalmologist and offers a self-assessment of visual function. The app is considered useful because research showed that a high percentage had diabetic retinopathy and other risk factors, so to consult ophthalmologist was necessary
Paper 64: Preferences for a Mobile HIV Prevention App for Men Who Have Sex With Men	Goldenberg et al. [74]	The paper describes how it should be an application that would promote the homosexual population to participate in HIV prevention. To do this, several focus group discussions were conducted (n = 38). It was evidenced that apps should have innovative ideas, educate and engage men to motivate them to use the application. Feeling safe and have the confidence was important, so the importance of protecting the privacy language and stressed was highlighted. To achieve adherence to the App, it should be easy to use, attractive and pushing homosexuals to concern about their safety
Paper 65: Randomized, controlled pilot trial of a smartphone app for smoking Cessation using Acceptance and commitment therapy	Bricker et al. [39]	The paper shows a comparative study of two mobile applications to quit smoking (“SmartQuit” and “QuitGuide”) based on different approaches. SmartQuit users open the application an average of 37 times versus 15 times from QuitGuide users. The quit rate was 13 % vs. 5 %. The authors indicate that the experimental design and low sample size prevents extrapolate the effectiveness of Apps
Paper 66: Sexual and reproductive health for young adults in Colombia: teleconsultation using mobile devices	Lopez et al. [22]	This document describes the research with 58 young people and adults in Bogota, Colombia. The study is aimed to evaluate the use of an app focused on the resolution of questions on health, with pre- and post-intervention survey. The main topics consulted were risks that they take not using a condom during sex, consumption of psychoactive drugs and lack of knowledge about sexually transmitted diseases. The results indicate that the strategy proposed was well accepted by young people. Although no significant result was evidenced, it is stated that the strategy proposed was well accepted by young people
Paper 67: Smartphone breast applications—What’s the evidence?	Mobasheri et al. [75]	The paper analyzed 185 applications focused on pathologies and breast problems. A large disconnect between the development of Apps and health professionals, and a marked lack of scientific evidence was showed. A review of the information provided is recommended, as well as add bibliographic references and mention the authors
Paper 68: Study of the usability of spaced retrieval Exercise using mobile devices for Alzheimer’s disease rehabilitation	Zmily et al. [76]	The research consists in a clinical-trial with 10 participants in early stages of Alzheimer’s, to whom was offering them two versions of the App “ADcope”, to enhance their abilities to perform activities of daily living and to promote their independence and social participation. The results are better in version based on images; although there was a good adaptation to the use and satisfaction of participants in both of them
Paper 69: Supporting cancer patients in illness management: usability evaluation of a mobile app	Mirkovic et al. [77]	This study is focused on the analysis of design, functionality and usability of a prototype App “Mobile Connect”, which is presented as a support to 7 cancer patients in managing their health, encouraging communication, management of symptoms, decision making, etc. Evidenced usability was very good (72 %). Researches also evidenced that to take into account the needs of users for developing this type of applications must be prerequisite for its development
Paper 70: The current status of mHealth for diabetes: will it be the next big thing?	Klonoff [78]	This paper presents a review of the role of mHealth in diabetes, presenting it as a value resource to monitoring diabetic patients through applications with glycemic control, which can be added to monitor other values. Researchers discussed that apps facilitate the monitoring and planning of personal goals, enabling better patient outcomes. However, lack of research, quality and rigour, as well as the need to overcome barriers such as privacy, the clinical benefit and economic returns, make difficult to give them a clinical use
Paper 71: The development of a mobile monitoring and feedback tool to stimulate physical activity of people with a chronic disease in primary care: a user-centered design	van der Weegen et al. [79]	This paper describes the development of an application to monitor an increase physical activity in chronic patients. 15 patients and 16 health professionals were involved in the research. It emphasizes the need to develop apps under an user-centred model. No clinical results are shown
Paper 72: The schisto track: a system for gathering and monitoring epidemiological surveys by connecting geographical information systems in real time	Leal Neto et al. [80]	The document shows us the development and main features of the app “Schisto Track”, created to facilitate the data collection on schistosomiasis. The app proved to be helpful to automate the activity, organizing data, retrieving, etc., so their use is recommended. In addition, the text describes how two types of access were used. A general access for users and other restricted to researchers involved in the project, ensuring that the database had information safe and confinable
Paper 73: Usage of multilingual mobile translation applications in clinical settings	Albrecht et al. [81]	Effectiveness “Xpromt” application as a tool to multilanguage support through interaction with the patient through a mobile device is evaluated in this research. Researchers offered the application to 39 health professionals. The results showed that, despite positively assess translation applications for communication with patients, there was no enthusiasm for “Xprompt”. While experts had clear expectations, users will not see benefit over time to be used in clinical settings. Finally, the authors remark the importance of assessing the degree of user acceptance for the development of these apps
Paper 74: User Preferences and Design Recommendations for an mHealth App to Promote Cystic Fibrosis Self-Management	Hilliard et al. [82]	The study attempts to define the characteristics that an app for adolescents with cystic fibrosis must have, for which the researchers combined qualitative and quantitative techniques in 16 participants. They expressed that access to information, automate the prescription process, and encourage communication with the staff care and family using the app, are very important resources that could be present in the app. It was also assessed positively the creation of a social network to bring together people and families in order to share experiences
Paper 75: Weight loss-there is an app for that! But does it adhere to evidence-informed practices?	Breton et al. [84]	The paper reviews and summarizes the content of the applications available on iTunes in 2009 (204), according to 13 practical for weight control based on scientific evidence. No application included the 13 practices. They found much variability regarding the number of practices, including 2 of them (daily food and weight evaluation) in 30 % of the apps. The most frequent resources were the interactive tool, nutritional data bases and educational material.
Paper 76: Who Uses Smoking Cessation Apps? A Feasibility Study Across Three Countries via Smartphones	BinDhim et al. [85]	The paper presents a comparative study of the characteristics of 602 users of “Quit Advisor”. This is an application to quit smoking. The research was carried out during 1 year in Australia, United Kingdom and United States. No significant differences in the average age of users and attempts to quit smoking between countries were found, but differences by gender and stage of change was evidenced. 77.5 % of users who had used previously other apps health, expressed do not take into account the credibility of the developer
Paper 77: Dietary Approaches to Stop Hypertension: Lessons Learned From a Case Study on the Development of an mHealth Behavior Change System	Mann et al. [88]	The study describes the development of an application of behavioural reducing hypertension based on the DASH diet. It takes into account the multimedia content, wireless devices (which can connect to the app), security and usability. The article concludes with 10 lessons learned during the development of the application, which highlights the need to break with tradition, the importance of a multidisciplinary approach, the platform features, the prototype must be flexible and scalable and the fact of that it is advisable to contact technological trade experts to grow through investors
Paper 78: Daily mood ratings via text message as a proxy for clinic based depression assessment	Aguilera et al. [89]	The aim of this research was to determine whether the information derived from the ratings of mood through SMS could serve as a reliable indicator for assessing the mood in clinical settings. 33 patients with depression were studied. As a control the PHQ-9 (Patient Health Questionnaire-9) was used. The results show that the SMS could fulfill this role
Paper 79: Incorporating customer empowerment in mobile health	Almunawara et al. [90]	This paper presents a health model that incorporates client empowerment in the mHealth in three dimensions: personal, social and medical. In addition, a survey was conducted to 366 individuals to assess their perception of the subject, as well as requirements for the model. Researchers concluded that the model integrated the widest scope of empowerment in the three proposed areas, serving as a guide to extend the empowerment of customers through mHealth
Paper 80: Anytime, anywhere access to secure, privacy-aware healthcare services: Issues, approaches and challenges	Anwar et al. [91]	This paper consists in a review aimed to rationalize and explore safety issues related to privacy in the mHealth. The authors examine existing approaches, addressing issues such as institutional infrastructure and government and policy challenges among countries to address the issues of security and privacy
Paper 81: Clinical laboratory data: acquire, analyze, communicate, liberate	Azzazy and Elbehery [92]	This review focuses on the use of smartphones to acquire, analyze, communicate, and deliver clinical laboratory data. The paper concludes that this resource can dramatically improve the quality and quantity of health assistance offered in the areas of limited resources
Paper 82: Photograph-based diagnosis of burns in patients with dark-skin types: The importance of case and assessor characteristics	Boissin et al. [93]	This study evaluated whether photographs of burns taken with a mobile from patients with darker skin types, could be used for diagnosis. 21 cases of varying complexity were studied and conducted a survey through the web. It was concluded that the size and depth of burns in these patients could be evaluated with photographs, at least as well as with the simple view in clinical settings, since the average accuracy rates were 67.5 and 66.0 % respectively
Paper 83: Mobile Health: empowering patients and driving change	Bradway et al. [94]	This document, after analyzing (literature review) the development of mHealth in the field of diabetes, concludes that although there has been good progress in apps for this medical condition, only a few of them have been validated and recognized to be used as tools for self-management of the disease. Therefore stresses the need to propose measures including the creation of a protocol to choose and use these tools or to establish financing plans and sustainability
Paper 84: Electronic personal maternity records: Both weband smartphone services	Chang et al. [95]	This paper presents a multi-platform information system (web and smartphones) to help women during pregnancy. 5 developers evaluated the usability through heuristics model of Nielsen, and 68 pregnant women completed a satisfaction survey. 80.9 % of respondents stated that the system was useful for controlling pregnancy
Paper 85: From black box to toolbox: Outlining device functionality, engagement activities, and the pervasive information architecture of mHealth interventions	Danaher et al. [96]	This review examines two important aspects in the design of mHealth interventions: mobile device functionality and the data architecture, which determines its ability to bring information to mobile phones, PCs and other devices. They conclude that mHealth interventions developers should work on these aspects to engage participants and promote the desired change in behaviour to achieve health
Paper 86: BP here, there, and everywhere – mobile health applications (apps) and hypertension care	Green [97]	Editorial referred to the promising potential of Apps health surveillance, monitoring and management of blood pressure in hypertensive patients is done
Paper 87: Impact of Mobile Diabetes Self-Care System on patients’ knowledge, behavior and efficacy	Guo et al. [98]	In this research, a system called “Mobile Diabetes Self-Care System”, for diabetes type 2 was evaluated. The system was created to facilitate patients to improve their capacity for self-care and trying to be flexible in time, place and developed options use. The effectiveness of the system was assessed in 28 individuals who completed questionnaires before and after the intervention. The results indicated that the mobile system had improved the knowledge and behaviour of patient self-management by 17 % and 22 %, respectively
Paper 88: Apps for life change: Critical review and solution directions	Helf and Hlavacs [99]	This review is focused on the current lack of scientific use in application development, inefficient and selective incorporation of gamification, low levels of customization and potential privacy and trust. Due to the multidisciplinary nature of this set of problems, the text proposes frameworks integrated and user-centered as the best solution.
Paper 89: Opportunities and barriers in service delivery through mobile phones (mHealth) for Severe Mental Illnesses in Rajasthan, India: A multi-site study	Jain et al. [100]	The document shows information about availability, usage patterns, barriers to mobile technology and felt needs for mental health services for patients with mental illness in the region of Rajasthan (India). 72 patients were interviewed. The preferred mode for delivery of service was through calls, telephone help lines for crisis resolution and follow-up of stable patients. The text concludes that the use of mobile technology has great potential for these patients.
Paper 90: Legal, Regulatory, and Risk Management Issues in the Use of Technology to Deliver Mental Health Care	Kramer et al. [101]	This review focuses on risk management related to mobile health applications and the use of social networks to provide services “Telemental Health” (TMH), resolving some concerns about the risks and developing a framework to effectively manage the risks associated with caring through TMH. The authors conclude that while it is important to be aware of these issues, the general principles to provide good clinical care, are the use of standards of practice set for the field of mental health, remain the core of the safe and effective practice
Paper 91: A content analysis of smartphone–based applications for hypertension management	Kumar et al. [102]	The study examines the quality and characteristics of apps focused on the control of hypertension (HTA). The vast majority offered tracking figures of blood pressure, weight or body mass index. It is concluded that consumers have a strong tendency to download and evaluate favourably applications without validation, so the need for greater supervision is evident in the development of medical applications for hypertension, especially when displayed in stores classified as medical devices
Paper 92: The lifestylisation of healthcare? ‘Consumer genomics’ and mobile health as technologies for healthy lifestyle	Lucivero and Prainsack [103]	This review highlights the challenge posed by “technologies for healthy living,” because it is an ambiguous space between the medical field highly regulated and the consumer market, less regulated. It is defined as the situation leads to new ways to create (and make sense) knowledge related to health. It also recognizes the enormous potential of these technological advances
Paper 93: NOPA, Usability testing of an application to help patients during th treatment of infectious, and chronic diseases in Brazil	Rodrigues et al. [104]	This document aims to present usability testing (through telephone interviews and focus groups) of a mobile application called NOPA to help patients during treatment of chronic diseases in Brazil. 10 individuals participated. The font size, the ability to send messages and participant status (online/offline) were not easy to identify in the App. The access to the application, to Find/Display the time of medication, and turn off the alarm to confirm that the drug had been taken, were the most valued features
Paper 94: Feasibility of a lifestyle intervention for overweight/obese endometrial and breast cancer survivors using an interactive mobile application	McCarroll et al. [105]	The study aimed to evaluate an intervention in 50 women survivors of endometrial or breast cancer with overweight or obese based on web (for contact with health professionals) and the “LoseIt” application. Results indicated that intervention by modification of lifestyle offered through the web and with the support of the App is a viable short term option to reduce the weight
Paper 95: Ergonomic evaluation and design of a mobile application for maternal and infant health for smartphone users among lower-income class Filipinos	Nocum et al. [106]	The paper presents a preliminary investigation to assess, using ISO 9126, a mobile application focused on disseminating relevant information to low-income pregnant women in the Philippines. Using the data collected, the researchers developed a new application (“Uyayi”) that provides all the information requested and takes into account the functionality, reliability and ease of use application designed for this group
Paper 96: Exploiting the potential and facing the challenges of mobile devices: application examples	Nunes and Simoes-Marques [107]	The document contents a review about apps related to health, from those used during leisure time until those for productivity time, trying to demonstrate their innovative potential, the analysis of the opportunities and remarking the challenges they represent
Paper 97: BPH laboratories: A proof-of-concept case on integrating smartphone diagnostics into clinical systems	Olla et al. [108]	The document presents through a proof of concept an App (“Immediate -IDA- Diagnostics and Analytics”), how it can be used to acquire, analyze and transmit laboratory data from a pre-surgical hospital module to any system information. The result shown that the use of the App could be convenient to read results of a pregnancy test in a pre-surgical room in real time and transmit it to electronic health records. Finally, the text concludes that it is a useful tool for decision making in real time, improving patient safety and effectiveness attention
Paper 98: Phone-based Intervention under Nurse Guidance after Stroke: Concept for Lowering Blood Pressure after Stroke in Sub-Saharan Africa	Ovbiagele [109]	This paper proposes a procedure called “Phone-based Intervention under Nurse Guidance after Stroke (PINGS)” for self-management of blood pressure in patients who have suffered a stroke and are poorly controlled in sub-Saharan Africa. PINGS could include the implementation of a program led by nurses to the use and control technology (custom text messages and tele-monitoring at home) aimed at increasing motivation and self-care and increase adherence to antihypertensive therapy
Paper 99: Enhanced healthcare personnel rostering solution using mobiletechnologies	Paschou et al. [110]	This document refers to the validation in India and Nepal, with a greater sample (122 cases), from an app previously designed and tested in a pilot test The App intended to be an instrument to differentiate epileptic attacks from those not being by medical staff. Different degrees of clinical evidence were evidenced as well as high sensitivity values and specificity; for this reason its use and check ups is recommended in other places
Paper 100: Validation of a phone app for epilepsy diagnosis in India and Nepal	Patterson et al. [111]	This document refers to the validation in India and Nepal, with a greater sample (122 cases), from an app previously designed and tested in a pilot test The App intended to be an instrument to differentiate epileptic attacks from those not being by medical staff. Different degrees of clinical evidence were evidenced as well as high sensitivity values and specificity; for this reason its use and check ups is recommended in other places
Paper 101: Review and analysis of existing mobile phone applications for health care associated infection prevention	Schnall and Iribarren [112]	The objective of this revision was to identify and provide a general vision of the apps available to support the prevention of hospital infections and to evaluate its functionality and potential uses in the clinical attention. 17 apps were studied. It was concluded that these could help to reduce the hospital infections, but due to the lack of available applications and functionality of the apps founded, it is required a greater development in its field
Paper 102: Mobile-health: A review of current state in 2015	Silva et al. [113]	This article presents an exhaustive revision of the state of the technique in the field of mHealth. The most important works published were studied, as well as the services of mHealth and the apps suggested by the industry. It was also approached the legislation and the necessity to define a paradigm of mHealth in the European Union and the United States. Finally, open and challenging issues were proposed about new solutions of mHealth for future works
Paper 103: The CAIDE Dementia Risk Score App: The development of an evidence-based mobile application to predict the risk of dementia	Sindi et al. [114]	The work presents the app “CAIDE Risk Score App” based in the toolkit (Cardiovascular Risk Factors, Aging, and Incidence of Dementia) to calculate the risk to suffer dementia. No results of validation were showed, taking into account that, in the moment of the development of the article, there were more than 700 downloads
Paper 104: Online Filipino-Americans’ perspectives on informatics-enabled health management	Thompson and Valdez [115]	The purpose of this study was to understand the perception of the perspectives of the Filipino-Americans about three types of information technology used for health management. An online poll was administered to 87 participants through Facebook. The sample was more likely to use social networks (SNS), except the mobile apps (mHealth) and hardly ever the personal health records were used (PHR). The participants informed about the advantages and disadvantages of using this kind of technology for the management of health (accessibility, credibility, privacy, ease of use and utility, among them)
Paper 105: Sexual and reproductive health and rights and mHealth in policy and practice in South Africa	Waldman and Stevens [116]	This article explores from a qualitative methodology (interviews to key informants and three case studies), the intersections between mHealth, health and the rights. It is showed that the degree in which mHealth tackles all the range of reproductive justice and the health and sexual and reproductive rights is limited, especially dealing with the government initiatives. The document maintains that the projects mHealth tend to avoid the controversial aspects of sexual health, whereas it is developed in favourable issues such as pregnancy and maternity
Paper 106: Acceptability of mobile health interventions to reduce inactivity-related health risk in central Pennsylvania adults	Yang et al. [117]	The aim of this study was to typify the need and the acceptability of mobile health interventions of behaviour among the rural adults with sedentary lives in Pennsylvania and to evaluate the interest and the value of the interventions based in mobile apps. 258 participants with Smartphones filled a brief survey thorugh Internet in October–November 2012. It was evidenced that most of the surveyed would use a free app or a low cost app to modify risk behaviours

However, the number of citations received of these articles is relatively small and widely dispersed. The sample shows an average of 6.35 (±13,56) references, excluding 2015 and 4.71 (±8.77) counting this year. Two papers highlight with 74 [12] and 63 [13] citations, facing many that have never been referenced. Interestingly, these two articles were published in 2011 and 2012 (respectively), when the exponential growth in the number of publications on mHealth began. Thus, we can find just 2 papers in 2011 (1.9 % of total found) while we locate 45 in 2014 (42.5 %). Similarly, only four articles were found in 2012 (3.8 %) and 26 in 2013 (24.8 %). Until October of 2015 we located 29 (27.4 %) papers published in 2015. In 2010 there were no relevant papers included in the search. This could be a sure sign that the scientific community is increasingly aware of the potential and the need to focus some research into this field.

Similarly, the mean number of citations received for years (excluding 2015), shows a clear downward trend (F = 36,72, 3 df; p < 0.001). As the years pass, the average is down from the 62 items on average in 2011 till 1.53 in 2014. Temporal matters could explain this, i.e., it stands to reason that more recent articles have had less time to be referenced (and that is the main reason because we decided to exclude 2015 from this analysis), but it is a trend that is also seen in 2012 (with an average of 23 references) and 2013 (7.85). Post hoc tests found significant differences between every year (p < 0.01).

**Fig. 3:**
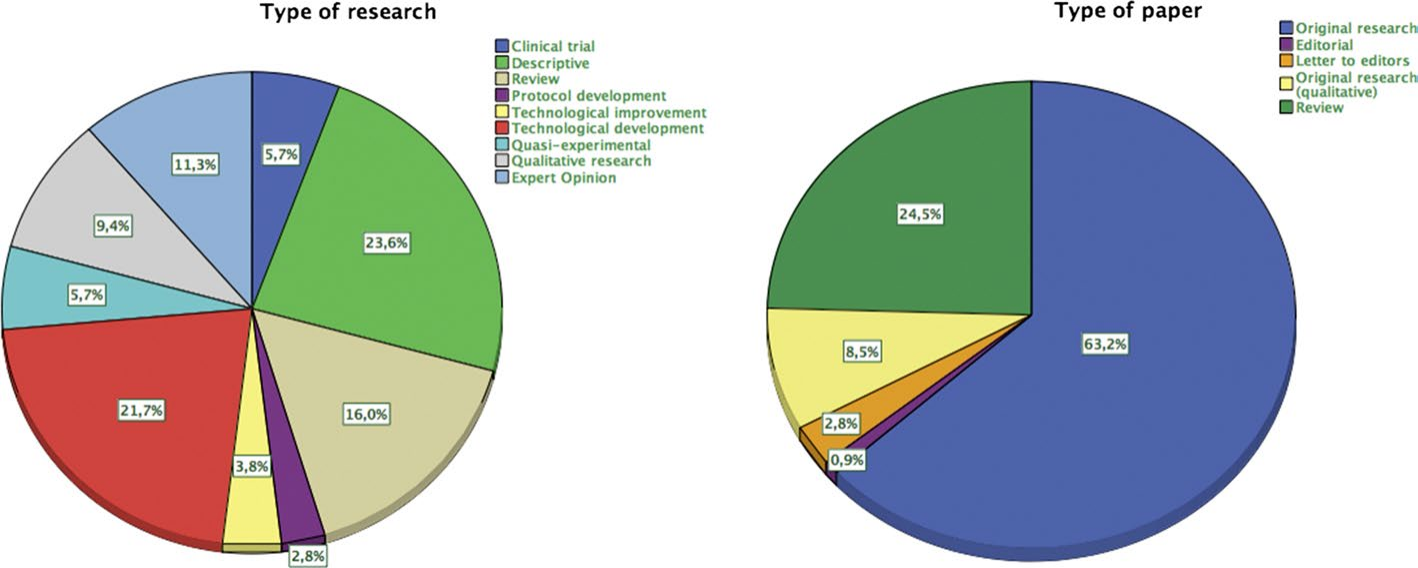
Type of research

In addition, considering the types of papers, we found that original researches are more prevalent (63.2 %), Besides this, we evaluated the type of the research carried out. We found descriptive studies (23.6 %), researches focused on technological development (21.7 %) and reviews (16 %) highlighting over the rest (see Fig. 3). This fact happens because most of the articles are focused on the user data collection and evaluation from apps designed to assess adherence or modification of habits to healthier lifestyles (studies with larger sample sizes). Another percentage of the original researches is represented by small pilot studies or clinical trials with smaller sample sizes (5.7 %). As we mentioned above, reviews are also frequent, something justifiable given the exponential growth of publications. This situation force researchers to conduct periodically synthesis and evaluation of trends in the development of apps and main findings, as well as the errors most frequently committed (especially as the methodological design is concerned). Another significant point was to find nine papers from qualitative research which represent 9.4 % of the types of research. It is understandable if we comprehend that this type of studies is very helpful in trying to expose the different realities of the new or unknown phenomena which we have a very little information or experience. A good example could be the expected results of interactions between mHealth apps and populations who want to improve their health. Furthermore, we were surprised because two of these articles had been published in computer science journals, where papers highly focused on applied and/or quantitative research are traditionally accepted. No statistically significant relation between the type of research and the number of citations received (p = 0.297) were found. Similarly, the type of research and the impact factor of the journal where the article is published did not show to be related (p = 0.094).

**Fig. 4:**
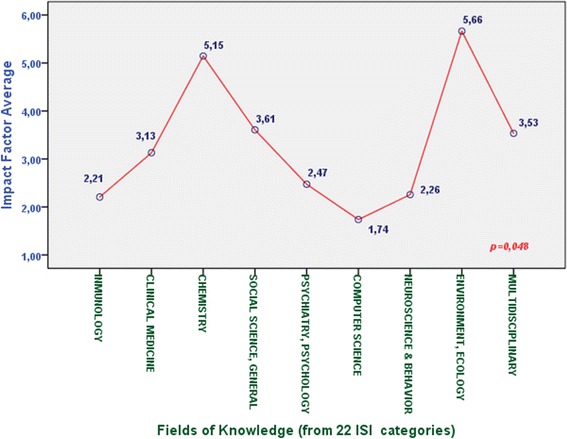
Impact factor average related to ISI 22 Fields

We also wanted to know the link between the field of knowledge where the publishing journal is classified and the impact factor (see Fig. 4). We found a clear relation (p < 0.05), highlighting “Environment/Ecology”, “Chemistry” and “Multidisciplinary” with higher average of impact factor. In any case, it is very important to evidence how fields of knowledge, very far from health, a priori, accept papers focused on mHealth, which, from our point of view, demonstrate the deep impact that this new topic is generating in many fields of knowledge, related or not to health sciences.

### Research teams and the role of interdisciplinarity

**Fig. 5:**
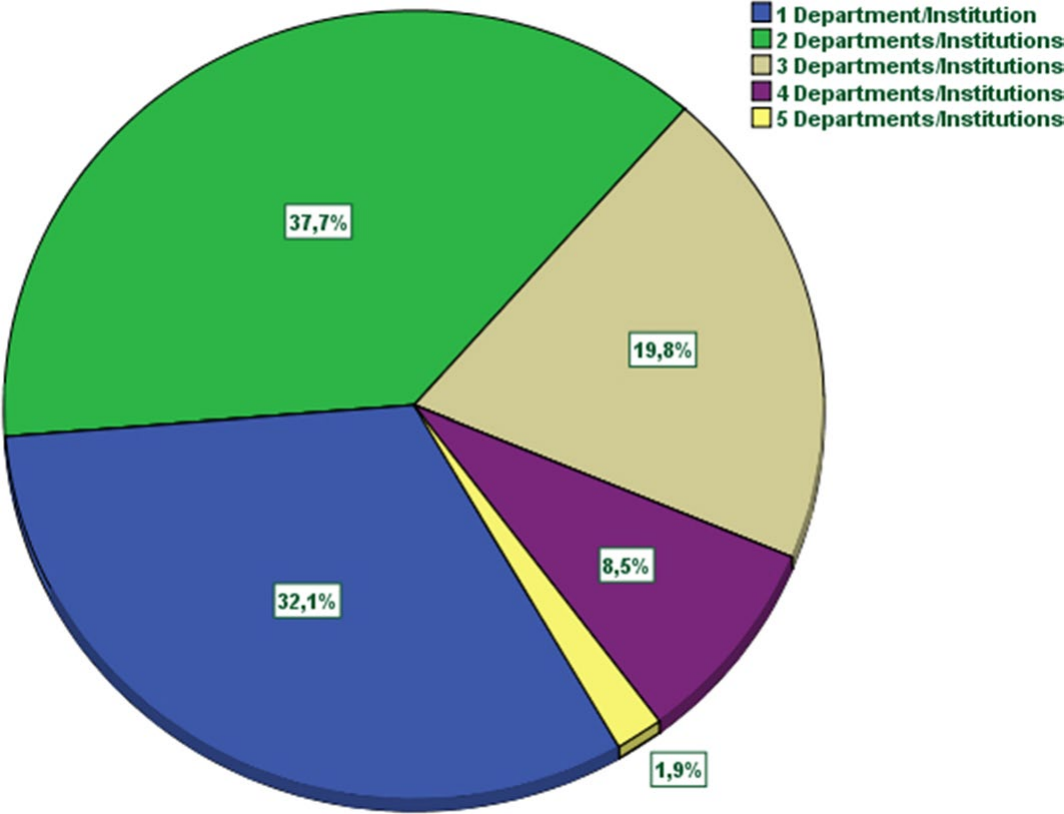
Number or departments/institutions focused on different areas involved in the research

The results show that more than half of the reviewed papers (67.9 %) had the participation of at least two departments/institutions focused on different topics, reaching the maximum amount of five different topics (see Fig. 5). Only 32.1 % [13–41] of the reviewed papers had participants focused on just one topic.

Besides this, we considered the interdisciplinarity, calculating the degree of diversity through Rao–Stirling (multidimensional index which includes the analysis of variety, balance and disparity). We found a very low and dispersed rates (0.07 ± 0.05). From our point of view, and related to the different topics where the departments involved in each paper are focused, these results mean that, although the diversity of the participants could seem high because they belong to different areas, finally they do not use the specific knowledge of each one, finding many references in each paper that belong to the same WoS categories (causing a low Rao–Stirling diversity).

### About interdisciplinary research teams, interdisciplinary publications, impact factor and number of citations received

**Fig. 6:**
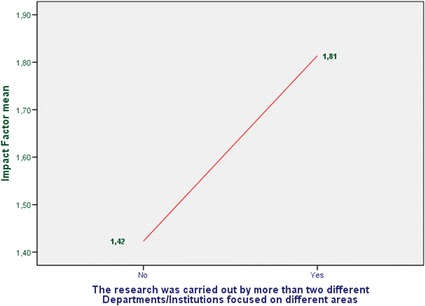
Relation between teams of more than 2 different departments/institutions focused on different areas and impact factor

**Fig. 7:**
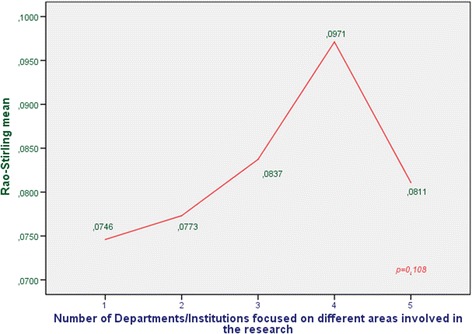
Relation between number of departments/institutions involved in the research and Rao–Stirling diversity

Although the visual examination of Fig. 6 shows a clear tendency for researches carried out by more than two departments/institutions from other fields of knowledge, are published in major journals, statistical analysis shows that these differences are not significant (p = 0.33), obtaining an average impact factor of 1.81 (±1.93) for researches carried out by more than two teams focusing on different fields, versus 1.42 (±1.88) for teams with two or fewer different fields of knowledge. However, we evidenced a light but positive and significant relation between the Rao–Stirling diversity and the impact factor (r = 0.182; p = 0.031) which means that a higher degree of interdisciplinarity increase of impact factor of the paper. Anyway, as we mentioned above, it is important to remember that the participation of multidisciplinary research teams does not necessarily means the presence of interdisciplinarity. This fact is supported by the analysis of the relation between the number of departments focused on different research areas for each paper and the value of Rao–Stirling diversity. In this case, we can not assume that the participation of research teams from different fields lead performing a multidisciplinary study. (p = 0.108) (see Fig. 7).

Another method to evaluate the impact of a paper consists in evaluating the number of citations received. Neither the Rao–Stirling (p = 0.063), the field where the publishing journal is classified according to 22 ISI web of knowledge categories (p = 0.811), or the number of departments focused on different fields of knowledge (p = 0.869), seem to be related to the number of citations received. The average number of citations for teams with more than two departments focused on different fields of knowledge is 6.52 (±9.9), versus an average of 6.14 (±9.6) citations for teams composed of two or less. From our point of view, this could be explained by the novelty of the research on mHealth, which seems to take precedence over the quality or focus of each investigation. We think that this could be also confirmed by the absence of statistical relations between the impact factor of the journals in which the papers were published, the number of citations received and the research design. That is, it would be logical to think that clinical trials, technological developments, and other quasi-experimental studies with higher levels of evidence, should be published in major journals or be more referenced. However, the result described above, indicates that this type of research designs is not better valued by publishers or other research investigators than others with lower levels of evidence (descriptive studies, qualitative researches, etc.).

**Fig. 8:**
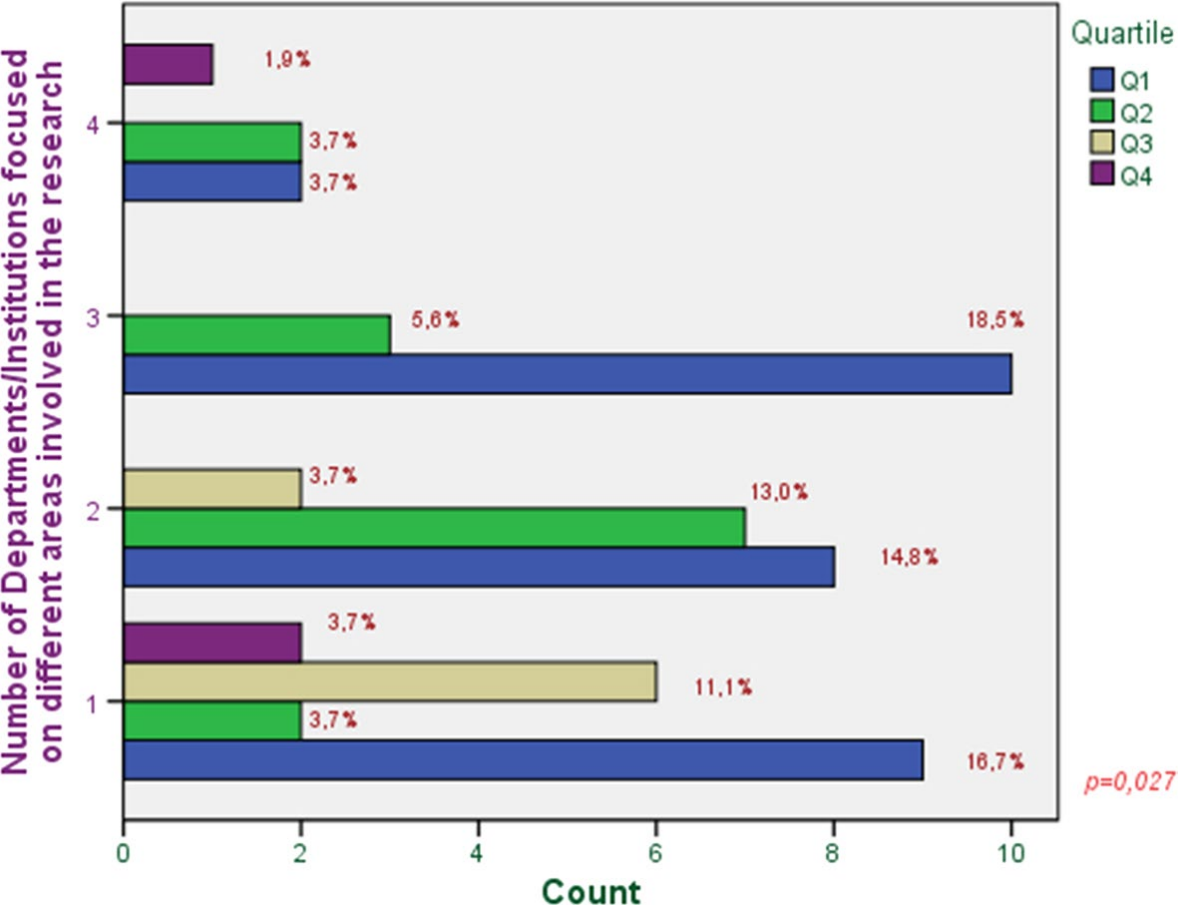
Relation between the quartile of publication and the number of departments/institutions focused in different areas involved in the research

A priori, it would seem that the interdisciplinary nature of the research teams is directly linked with access to publication in journals indexed in higher quartiles. This fact is confirmed by finding significant relations (p = 0.027) between the quartile of the journal in which the articles were published and the number of departments focused on different areas involved in the investigation (see Fig. 8). By contrast, we did not find relation with Rao–Stirling diversity (p = 0.475); showing that the fact of accessing higher quartiles may be related to the presence of multidisciplinary teams in the investigation, which does not necessarily mean that researches show an interdisciplinary approach.

Another aspect to take into account is referent to the number of articles that are included in our study, which covers until October of 2015. Because of that, it is difficult to stablish the progression in the number of papers in this year. We considered that the number of papers in the last quarter of this year (2015) continue with a increasing tendency, but, it seems that in a lineal level (not exponential as in previous years). This fact could be only estimated in studies that will be carried out in 2016.

### Limitations

The results should be taken with caution given various limitations. In this sense, Yadros et al. [6] stated some limitations as: “the inaccuracies in the WoS categories used to define subdisciplinary categories may create biases in the indicators of citation impact (since citation impact is highly affected by normalisation) and may have an important effect as well in diversity measures.”, or “the inclusion or not of some control variables such as number of co-authors, institutions or article length is open to debate and these may have an effect on results.”, among others.

On the other hand, from the revised bibliography it is possible to extract that for calculating certain bibliometric indexes (as Rao–Stirling diversity), it is usual to consider a higher quantity of the number of papers than the number of them that we have considered in this work. This fact is because the main objective in our paper was to realize a systematic review and no the calculation of bibliometric indexes. The number of revised papers is considerably minor, so, it would be advisable to take with caution the results of this bibliometric index.

## Conclusions

There are mHealth papers of all kinds (trials, analytical, descriptive, reviews, etc.) and its number grows at an exponential rate. This could show how technologies related to mHealth are reaching the scientific field. These technologies are reaching the population even faster. Therefore, mHealth is becoming an interesting research topic in both fields, health and computer sciences (hasta 2014 siendo más moderado en 2015).

These articles are published in high impact journals. We have found them in specific journals (focused on eHealth) and in generalist health and computing journals. Generalist journals have just begun to accept research based on the application of mHealth technologies. This highlights the growing importance of this topic.

There are already studies that warn of methodological deficits in some studies in mHealth, low accuracy and no reproducibility. Studies of low precision and poor reproducibility, coupled with the low evidence, provide low degrees of recommendation of the interventions targeted and therefore low applicability. The market apps, mostly lack of scientific evidence or participation of health professionals. This should compel publishers and researchers to be more stringent on the design of experiments and in the publication of results.

Fundamentally, it is possible to extract two main conclusions from our study. First, it is evident the increased interest that publishers of scientific journals are showing to mHealth, given the steady increase in publications focusing on this issue, regardless of the subject area. Second, it seems clear that the participation of multidisciplinary teams (with a variety of professionals focused on different areas of knowledge) is not necessarily linked to the presence of interdisciplinary approaches. Finally, this interdisciplinarity plays a limited role in the impact of the papers (measured by the impact factor of the journal where they are published and the number of citations that they received), this fact could be more related to the novelty of the topic of research.
